# Synthesis and preliminary assessment of the anticancer and Wnt/β-catenin inhibitory activity of small amide libraries of fenamates and profens

**DOI:** 10.1007/s00044-017-2001-z

**Published:** 2017-08-05

**Authors:** Bini Mathew, Judith V. Hobrath, Wenyan Lu, Yonghe Li, Robert C. Reynolds

**Affiliations:** 1 Drug Discovery Division, Southern Research Institute, 2000 Ninth Avenue South, Birmingham, AL 35205 USA; 2 Drug Discovery Unit, College of Life Sciences, University of Dundee, Dundee, DD1 5EH UK; 3 Division of Hematology and Oncology, The University of Alabama at Birmingham, Birmingham, Alabama 35294 USA

**Keywords:** NSAIDs, Fenamates, Amides, Wnt/β-catenin, Cancer

## Abstract

As part of an ongoing program to study the anticancer activity of non-steroidal anti-inflammatory drugs (NSAIDs) through generating diversity libraries of multiple NSAID scaffolds, we synthesized a series of NSAID amide derivatives and screened these sets against three cancer cell lines (prostate, colon and breast) and Wnt/β-catenin signaling. The evaluated amide analog libraries show significant anticancer activity/cell proliferation inhibition, and specific members of the sets show inhibition of Wnt/β-catenin signaling.

## Introduction

Non-steroidal anti-inflammatory drugs (NSAIDs) are the most widely used class of drugs for the treatment of pain and inflammation. The anti-inflammatory mechanism of the NSAIDs is attributed to the inhibition of the cyclooxygenases (COXs) and reducing the synthesis of prostaglandin signaling molecules (Vane 1971). There are two major isoforms of the COXs, COX-1 and COX-2. COX-1 is constitutively expressed in most tissues and plays an important role in tissue homeostasis, while COX-2 is induced as part of the acute inflammatory pathway. Epidemiological, preclinical and clinical studies have demonstrated the chemopreventive efficacy of NSAIDs by reducing cancer incidence in the general population by up to 50% (Thun et al. 2002; Chan 2002; Reeder et al. 2004; Soh and Weinstein 2003). The depletion of physiologically important prostaglandins through chronic COX (COX-1 or COX-2) inhibition, however, can have significant life threatening side effects including gastrointestinal, renal, and cardiovascular toxicity (Cannon and Cannon 2012; Yu et al. 2012; Mukherjee 2002; Vane and Botting 1998; Vane et al. 1998). Unfortunately, these side effects limit the utility of NSAIDs for cancer chemoprevention, which tends to require high dosages and chronic treatment. NSAIDs are most commonly believed to display their anticancer effects through inhibition of COX-2, as this isozyme is thought to play a role in carcinogenesis and is often over expressed in human premalignant and malignant tissues (Brown and DuBois 2005; Husain et al. 2002; Eberhart et al. 1994). On the other hand, certain studies indicate that NSAIDs also promote apoptosis through mechanisms that are independent of COX inhibition. This proposition is further supported by the fact that compounds, structurally similar to NSAIDs but not significantly inhibiting COX isozymes, may have chemopreventive and proapoptotic properties (Piazza et al. 2010; Elder et al. 1997; Hanif et al. 1996; Alberts et al. 1995).

The Wnt/β-catenin signaling pathway is a crucial player in the management of cell proliferation, migration, and differentiation, thus making it a powerful regulator of embryonic development and tumorigenesis (Barker and Clevers 2006). Wnt proteins are secreted glycoproteins that bind to the low-density lipoprotein receptor-related protein5/6 and Frizzled to activate Wnt/β-catenin signaling. A large body of evidence suggests that there may be direct effects of NSAIDs on the Wnt/β-catenin signaling pathway (Giardiello et al. 1993; Koehne and DuBois 2004; Jolly et al. 2002; Yang et al. 2003; Mahmoud et al. 1998; Boon et al. 2004; Gala and Chan 2015; Egashira et al. 2017; Preisner et al. 2015; Sareddy et al. 2013; Stein et al. 2011; Lu et al. 2009; Bombardo et al. 2017). For example, it was found that aspirin and indomethacin attenuate the transcription of β-catenin/TCF-responsive genes, by modulating T-cell factor (TCF) activity without disrupting β-catenin/TCF complex formation (Dihlmann et al. 2001).

Fenamate NSAIDs, including tolfenamic, mefenamic and flufenamic acid have been derived from anthranilic acid, a close isosteric analog of salicylic acid. Salicylic acid is a metabolite of salicin and the active ingredient of the earliest anti-inflammatory herbal medicines, first isolated from willow bark. Interestingly, members of the NSAID fenamates and profens have been also shown effective for cancer prevention (Basha et al. 2011; Kang et al. 2012; Woo et al. 2004; Somchit et al. 2009; Lovering et al. 2004; Zhu et al. 1999; Mayorek et al. 2010; Marjanovic et al. 2007). We previously reported that a relatively simple alteration to sulindac in the form of sulindac sulfide amide (SSA) demonstrated excellent anticancer activity compared to the parent compound sulindac sulfide in vitro as well as having in vivo xenograft activity (Piazza et al. 2009). These findings inspired us to synthesize a series of libraries from other NSAID scaffolds, such as tolfenamic acid, mefenamic acid, flufenamic acid, diclofenac and fenoprofen, replacing the carboxylate with an amide functional group as in SSA in order to examine the anticancer activity of the amide libraries against three cancer cell lines (prostate, colon and breast). These compounds were further screened against Wnt/β-catenin signaling to elucidate COX-independent mechanisms of their effects.

## Materials and methods

### Chemistry

**Fig. 1:**
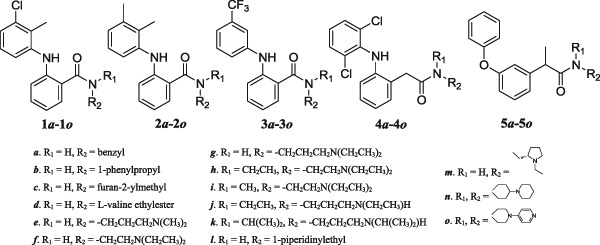
General structures and substitutions of synthetic libraries

Figure 1 shows the general structures of the amide diversity series: (**1**) tolfenamic amides; (**2**) mefenamic amides; (**3**) flufenamic amides; (**4**) diclofenac amides; and (**5**) and fenoprofen amides.

**Scheme 1:**
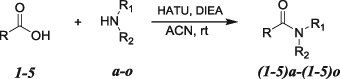
General synthetic scheme to prepare NSAID amides

These compounds were prepared from the corresponding commercially available fenamates and fenoprofen by coupling with an amine set (**a** *–* **o**) using HATU (Carpino 1993) as the amide coupling reagent to afford compounds (**1**–**5)a–(1**–**5)o** in good yields (Scheme 1).

### Biological studies

#### Quantitative high-throughput screen (qHTS) cell proliferation assays

All target compounds were screened against three cancer cell lines (prostate, colon and breast) using a qHTS format. In brief, liquid handling was performed on a Biomek FX with a 384-multichannel head. In 384 well plates, compounds were arrayed in columns 3–22 leaving 32 wells for positive and negative controls. All compounds were diluted together in a plate to plate transfer. Cells were then added to assay plates containing diluted compound using a Matrix/Thermo wellmate. Cells were incubated with compound for three cell doublings. Due to differences in growth rates between cell lines, the incubation period for PC3 and HT-29 cells was 72 h, but was increased to 96 h for MDA-MB-231 cells. Plates were incubated for the appropriate time (72 or 96 h) and cell viability was determined using Cell Titer Glo (Promega).

The dose response format employed a cross-plate method rather than an in-plate method, allowing for more efficient compound dilution and addition to assay plates. Two-fold dilutions of the compound mother plate were aliquoted to a series of 384-well plates using a stacked plate (or cross-plate) format. Object manager was used to create the assay plates by replicating the compound mother plate and assigning concentration values to the assay plates. Luminescence values were read on the envision plate reader for each of the assay plates. The entire experiment of assay plates was set up in a single day with a complete read of all plates occurring at 72 h and or at 96 h as required for the cell lines. Data were imported and analyzed within 24 h of the endpoint read. From set up to final report, all data points were generated and reported within one week. Therefore, only a single passage was required for each cell line, eliminating potential variation due to passage and cell count.

Data were analyzed using Activity Base software (IDBS). Data were imported directly into the database and calculated using an ActivityBase XE template where the Virtual Plate functionality was employed to maintain the link between the assay plates and the compound mother plate from which they were created. For each plate the median, standard deviations, coefficient of variations and *Z* values were calculated for the control wells. These values were used to assure quality and consistency across all test plates and to normalize percent cell viability for each well. XLFit and MathIQ were used within the ActivityBase XE template to plot the dose response curves and calculate CC_50_ values. The CC_50_s were calculated by plotting the cell viability relative to the mean of the cell control at each of the tested compound concentration. Compounds that caused cell viability <80% were considered active. Values were calculated only for active compounds using a 4-parameter Levenburg-Marquardt algorithm (XLFit #205), with the maximum and minimum locked at 0 and 100 respectively. Data and graphical results were then reported and compared across the three cell lines.

#### Wnt/β-catenin signaling screens

The effects of all target compounds on Wnt/β-catenin signaling were examined with the Wnt reporter luciferase assay in HEK293 cells as previously described (Lu et al. 2011; Lu et al. 2012). In brief, cells were plated into 24-well plates. After overnight culture, cells were transiently transfected with LRP6 plasmid (kindly provided by Dr. Christof Niehrs, Deutsches Krebsforschungszentrum, Heidelberg, Germany) along with the Wnt signaling reporter construct Super8XTOPFlash (kindly provided by Dr. Randall T. Moon, University of Washington, Seattle) and β-galactosidase-expressing vector (Promega) by FuGENE HD (Roche). After 24 h incubation, cells were treated with each individual compound at the indicated concentration. Cells were then lysed 24 h later and both luciferase and β-galactosidase activities were determined. The luciferase activity was normalized to the β-galactosidase activity. Initial activity was measured at 100 µM, and, typically for those compounds that showed <50% activity relative to the control level and cancer cell line inhibition, a dose of 10 µM was tested in order to determine if Wnt/β-catenin activity was dose-dependent.

## Results and discussion

### Screening results

Table 1 lists the anticancer activity of tolfenamic amide analogs **1** against colon, prostate, and breast cancer cell lines as well as their Wnt/β-catenin signaling data. Benzyl amide derivative of tolfenamic acid **1a** displayed significant anticancer activity in all the three assays. 1-Phenylpropyl amide **1b** exhibited moderate activity in colon, prostate and breast cancer assays with CC_50_ values of 15.92, 25.37 and 18.08 µM, respectively. Compound **1c** with a furan-2-ylmethyl group at the amide linker showed better inhibitory potency than **1b**, but was less potent than **1a**. Interestingly, compounds **1a**, **1b** and **1c** at 10 µM significantly inhibited Wnt/β-catenin signaling in HEK293 cells in a dose-dependent manner as signaling activity decreased at the higher dose of 100 µM, suggesting that the inhibition of Wnt/β-catenin signaling could contribute to anticancer activity.

**Table 1:** Anticancer and Wnt/β-catenin signaling data of tolfenamic amides **1a**–**1o**

Cpd	Cell Line - CC_50_ (µM)			Wnt/β-catenin	
	HT29	PC3	MDA-MB-231	% Control (100 µM)	% Control (10 µM)
**1a**	0.99 ± 0.35	5.89 ± 2.50	7.27 ± 6.61	4.78 ± 0.84	30.66 ± 2.88
**1b**	15.92 ± 2.14	25.37 ± 4.38	18.08 ± 4.64	13.23 ± 3.89	56.55 ± 3.55
**1c**	3.11 ± 0.49	9.43 ± 1.16	6.21 ± 3.20	8.40 ± 0.74	47.12 ± 4.65
**1d**	>50	>50	>50	51.15 ± 3.41	ND
**1e**	18.93 ± 1.29	26.42 ± 2.86	26.81 ± 1.98	66.61 ± 7.70	ND
**1f**	6.58 ± 0.58	7.92 ± 0.87	10.67 ± 0.85	N/A	148.21 ± 0.63
**1g**	8.74 ± 1.14	13.29 ± 1.56	16.57 ± 1.97	N/A	ND
**1h**	12.04 ± 1.24	35.05 ± 1.96	>50	69.26 ± 9.56	ND
**1i**	20.02 ± 1.83	>50	>50	157.05 ± 3.74	ND
**1j**	17.33 ± 1.64	>50	>50	81.87 ± 4.04	ND
**1k**	6.05 ± 0.58	14.51 ± 1.74	16.68 ± 1.82	N/A	128.71 ± 13.66
**1l**	7.15 ± 0.73	9.56 ± 0.83	10.39 ± 1.15	N/A	163.73 ± 7.58
**1m**	6.69 ± 0.43	8.19 ± 0.77	10.21 ± 0.93	N/A	179.39 ± 6.78
**1n**	11.31 ± 1.31	29.12 ± 3.22	21.11 ± 1.30	16.24 ± 2.39	129.82 ± 7.08
**1o**	5.43 ± 0.28	8.12 ± 0.89	14.15 ± 1.03	N/A	95.84 ± 0.30

*N/A* not available due to cell toxicity at this concentration, *ND* not done

The amino acid analog **1d** led to a complete loss of potency in the proliferation assays although modest activity was seen at 100 µM in the Wnt/β-catenin signaling assay. Acyclic basic amide derivatives of tolfenamic acid **1e–** **1k** displayed various levels of anticancer activity and CC_50_ values ranged from 6 µM to >50 µM. *N*,*N*-diethylethylamide derivative **1f** and *N*,*N*-diethylpropylamide derivative **1g** demonstrated similar activity against the three cell lines. The introduction of an ethyl or a methyl group at the amide nitrogen of **1f** decreased the inhibitory activity as shown by **1h** and **1i**. In the case of **1j**, replacement of one ethyl group at the terminal nitrogen with a hydrogen and introduction of an ethyl group at the amide nitrogen of **1g** reduced the inhibitory activity by two-fold in HT-29 cells. Replacing both ethyl groups of **1j** by two isopropyl groups (**1k**) improved the activity by three-fold. Compounds **1l**–**1o** are the cyclic basic amide analogs of tolfenamic acid. Among these four compounds, 1-piperidinylethyl analog **1l** and 1-ethylpyrrolidinylmethyl analog **1m** have similar activity and 4-pyridylpiperazine derivative **1o** displayed slightly better anticancer activity than the other three examples. Notably, compounds **1d**–**1o** displayed weak activity at best against Wnt/β-catenin signaling in HEK293 cells.

Screening data for the mefenamic amide series **2** are shown in Table 2. In general, compounds (**2a**–**2o**) showed modestly decreased cell growth inhibition potency compared to the tolfenamic amide series **1a**–**1o**. Among this series, benzyl amide derivative **2a**, 1-piperidinylethyl derivative **2l**, 1-ethylpyrrolidinylmethyl derivative **2m**, and 4-pyridylpiperazine derivative **2o** are the most active compounds with CC_50_ values ranging from 5  to 9 µM against the HT-29 cell line. Moreover, benzyl amide derivative **2a** also suppressed Wnt/β-catenin signaling in HEK293 cells with activity that was modestly less than the related tolfenamic amides **1a**. Similar results were seen with analog **2c** as compared to the related compound **1c**.

**Table 2:** Anticancer and Wnt/β-catenin signaling data of mefenamic amides **2a**–**2o**.

Cpd	Cell line - CC_50_ (µM)			Wnt/β-catenin	
	HT29	PC3	MDA-MB-231	% Control (100 µM)	% Control (10 µM)
**2a**	5.93 ± 0.95	17.69 ± 2.91	17.35 ± 9.25	8.15 ± 1.73	64.46 ± 0.36
**2b**	31.50 ± 3.63	>50	25.83 ± 3.97	42.15 ± 4.53	73.55 ± 3.55
**2c**	12.06 ± 1.21	33.91 ± 4.86	10.78 ± 2.65	2.25 ± 0.70	59.64 ± 7.64
**2d**	>50	>50	>50	47.19 ± 6.36	ND
**2e**	>50	>50	>50	255.95 ± 59.07	ND
**2f**	11.52 ± 0.88	20.55 ± 2.39	19.21 ± 2.08	N/A	163.22 ± 37.56
**2g**	15.14 ± 0.41	33.82 ± 4.77	27.44 ± 1.64	196.32 ± 9.61	ND
**2h**	19.68 ± 0.84	>50	>50	178.13 ± 9.46	ND
**2i**	39.45 ± 2.33	>50	>50	189.74 ± 10.96	ND
**2j**	28.55 ± 0.88	>50	>50	179.79 ± 15.34	ND
**2k**	12.98 ± 0.39	29.06 ± 2.98	30.81 ± 1.73	18.84 ± 2.24	ND
**2l**	6.08 ± 0.38	10.41 ± 1.29	11.32 ± 1.35	N/A	132.88 ± 10.51
**2m**	8.27 ± 0.45	9.82 ± 1.11	13.20 ± 0.43	N/A	174.42 ± 37.71
**2n**	19.51 ± 0.94	>50	>50	144.22 ± 7.82	ND
**2o**	8.73 ± 0.61	20.45 ± 2.55	>50	100.10 ± 8.28	ND

*N/A* not available due to cell toxicity at this concentration, *ND* not done

Our results for the flufenamic amides **3** are summarized in Table 3. The activity pattern of these compounds (**3a**–**3o**) is very similar to the mefenamic amide series **2a**–**2o**. Compounds **3a** (benzyl amide), **3f**(*N*,*N*-diethylethyl amide), **3k** (N-isopropylpropyl amide), **3** **l** (1-piperidinylethyl amide), **3m** (1-ethylpyrrolidinylmethyl amide) and **3o** (4-pyridylpiperazine amide) showed moderate activity in all the three assays. However, compounds **3a**–**3o** displayed modest to little activity against Wnt/β-catenin signaling in HEK293 cells although the amides **3a**–**3c** consistently were higher in activity as in tolfenamic amides (**1**) and mefenamic amide (**2**) series.

**Table 3:** Anticancer and Wnt/β-catenin signaling data of flufenamic amides **3a**–**3o**

Cpd	Cell Line - CC_50_ (µM)			Wnt/β-catenin	
	HT29	PC3	MDA-MB-231	% Control (100 µM)	% Control (10 µM)
**3a**	7.75 ± 1.29	19.79 ± 3.03	22.55 ± 9.86	4.56 ± 0.08	62.33 ± 7.32
**3b**	35.64 ± 4.04	>50	32.07 ± 3.34	9.10 ± 0.72	74.87 ± 8.54
**3c**	15.11 ± 1.17	27.38 ± 2.60	17.91 ± 3.61	6.89 ± 7.98	70.49 ± 8.01
**3d**	>50	>50	>50	33.31 ± 0.43	ND
**3e**	17.82 ± 0.73	41.33 ± 6.50	41.44 ± 2.07	152.99 ± 13.15	ND
**3f**	8.16 ± 0.48	15.45 ± 1.78	14.78 ± 1.25	N/A	ND
**3g**	16.58 ± 0.68	33.89 ± 2.63	29.88 ± 2.24	95.15 ± 7.59	ND
**3h**	23.55 ± 1.14	>50	>50	124.87 ± 3.64	ND
**3i**	39.92 ± 2.45	>50	>50	156.09 ± 3.79	ND
**3j**	38.76 ± 2.60	>50	>50	119.75 ± 5.61	ND
**3k**	10.63 ± 0.26	25.25 ± 2.98	24.12 ± 2.35	39.83 ± 5.51	ND
**3l**	7.06 ± 0.65	13.21 ± 1.69	15.71 ± 0.89	N/A	153.34 ± 8.53
**3m**	7.83 ± 0.35	10.44 ± 0.99	12.02 ± 0.53	N/A	131.68 ± 2.86
**3n**	22.82 ± 0.74	>50	>50	80.49 ± 6.54	ND
**3o**	11.17 ± 0.47	17.14 ± 1.45	30.39 ± 3.00	5.31 ± 1.92	ND

*N/A* not available due to cell toxicity at this concentration, *ND* not done

We next turned our attention to anticancer activity of diclofenac amides **4** **a**–**4o** (Table 4).

**Table 4:** Anticancer and Wnt/β-catenin signaling data of diclofenac amides **4a**–**4o**

Cpd	Cell Line - CC_50_ (µM)			Wnt/β-catenin	
	HT29	PC3	MDA-MB-231	% Control (100 µM)	% Control (10 µM)
**4a**	>50	31.82 ± 9.34	30.75 ± 7.76	131.26 ± 52.55	ND
**4b**	>50	>50	>50	15.97 ± 3.91	ND
**4c**	>50	35.47 ± 10.32	49.33 ± 6.53	52.36 ± 5.34	ND
**4d**	>50	32.82 ± 5.34	42.07 ± 7.57	13.51 ± 11.78	117.56 ± 4.50
**4e**	18.72 ± 1.29	>50	42.21 ± 4.98	132.99 ± 25.46	ND
**4f**	5.48 ± 0.20	16.87 ± 2.63	19.09 ± 2.52	N/A	200.60 ± 18.09
**4g**	7.52 ± 0.28	22.26 ± 2.00	18.71 ± 0.85	N/A	173.75 ± 4.36
**4h**	7.25 ± 0.28	14.26 ± 1.61	25.15 ± 2.68	N/A	163.92 ± 5.45
**4i**	8.85 ± 0.32	22.42 ± 2.00	29.67 ± 1.61	11.90 ± 7.28	151.45 ± 5.82
**4j**	7.21 ± 0.43	10.83 ± 1.58	19.16 ± 1.59	N/A	134.87 ± 13.58
**4k**	4.56 ± 0.15	6.65 ± 0.74	9.11 ± 0.32	N/A	138.80 ± 0.96
**4l**	6.29 ± 0.18	10.75 ± 1.25	14.97 ± 1.16	N/A	190.60 ± 14.46
**4m**	6.42 ± 0.57	13.17 ± 1.46	13.51 ± 1.12	N/A	186.88 ± 12.50
**4n**	10.15 ± 0.49	19.8 ± 2.34	16.64 ± 0.93	6.39 ± 1.66	ND
**4o**	3.51 ± 0.35	10.33 ± 1.72	16.57 ± 1.33	181.77 ± 61.37	ND

*N/A* not available due to cell toxicity at this concentration, *ND* not done

In this series, acyclic (**4e**–**4k**) and cyclic (**4l**–**4o**) basic amide compounds exhibited significant inhibitory activity against cancer. The activity of aromatic amides was relatively weak. Among the acyclic series, N-isopropylpropyl amide derivative **4k** displayed relatively potent activity against all three cell lines. Compound **4o**, with a 4-pyridylpiperazine group at the amide linker, was found to be more active than other compounds in the cyclic amide series. Notably, compounds **4a**–**4o** displayed no or weak activity against Wnt/β-catenin signaling in HEK293 cells.

We next examined the anticancer activity of corresponding fenoprofen analogs **5** (Table 5). None of these compounds, except N-isopropylpropyl amide analog **5k** and 4-pyridylpiperazine amide analog **5o**, exhibited good activity. Furthermore, compounds **5a**–**5o** displayed weak activity at best against Wnt/β-catenin signaling in HEK293 cells.

**Table 5:** Anticancer and Wnt/β-catenin signaling data of fenoprofen amides **5a**–**5o**

Cpd	Cell Line—CC_50_ (µM)			Wnt/β-catenin	
	HT29	PC3	MDA-MB-231	% Control (100 µM)	% Control (10 µM)
**5a**	>50	>50	>50	9.60 ± 1.54	109.27 ± 12.08
**5b**	>50	>50	>50	6.77 ± 0.39	114.35 ± 18.16
**5c**	>50	>50	>50	71.24 ± 10.54	ND
**5d**	>50	>50	>50	4.76 ± 43	79.12 ± 6.12
**5e**	>50	>50	>50	82.57 ± 4.03	ND
**5f**	29.6 ± 2.04	>50	>50	131.04 ± 5.43	ND
**5g**	35.72 ± 9.18	>50	>50	139.50 ± 13.04	ND
**5h**	15.07 ± 1.57	>50	>50	77.34 ± 5.76	116.65 ± 11.31
**5i**	23.11 ± 3.60	>50	>50	114.07 ± 18.30	ND
**5j**	17.57 ± 1.28	41.3 ± 3.34	>50	140.26 ± 18.54	ND
**5k**	7.46 ± 0.63	24.01 ± 3.84	30.8 ± 7.06	24.76 ± 4.86	131.90 ± 7.46
**5l**	20.37 ± 1.82	>50	>50	125.69 ± 7.98	ND
**5m**	28.51 ± 3.16	>50	>50	92.17 ± 8.49	ND
**5n**	13.83 ± 1.30	>50	>50	101.52 ± 10.31	ND
**5o**	7.9 ± 0.84	11.51 ± 0.90	13.22 ± 2.35	8.51 ± 1.30	82.33 ± 3.50

*ND* not done

### In silico evaluation of lipophilicity/physicochemical properties

Compounds that have attractive physicochemical properties, such as cell permeability and metabolic stability are more likely to succeed as lead candidates in early drop development. LogD and the molecular weight (MW) have been identified as properties showing correlation with permeability and stability data available for large data sets (Waring 2009; Johnson et al. 2009). Using Caco-2 permeability data for 16,227 compounds and human liver microsome (HLM) stability determined for 47,018 compounds, optimal ranges for logD and MW have been derived that describe compounds meeting both requirements, good permeability and HLM stability (Johnson et al. 2009). On a plot of MW versus logD these optimal ranges cover a triangular area with baseline at MW 200 and logD −2 to 5 and an apex at MW 450 between logD 1 to 2, referred to as the Golden Triangle area. Several analogs from each presented series map to the Golden Triangle area, identified with labeled data points in the MW versus logD plot (Supplemental materials), where logD values were computed using StarDrop (version 6.2.0). For example, these optimal ranges are satisfied by analogs with promising activities, such as **2m** (logD 2.77, MW 351.5), **5k** (logD 2.08, MW 382.5), **5o** (logD 2.66, MW 387.5). These compounds are predicted to possess optimal values associated with good permeability and metabolic stability properties. Metabolically labile site predictions included in Supplemental materials support that compound **5o** has minimal metabolic liabilities and predicts that it has a low estimated efficiency of CYP3A4 metabolism as computed based on all metabolic sites in the molecule. In the case of compound **5k**, a single labile site is predicted at the isopropylamine group suggesting redesign of this group to achieve enhanced metabolic stability for this scaffold. The remainder of labile sites in **5k** are predicted to be relatively stable or moderately stable to metabolism. Overall, both compounds **5o** and **5k** have reduced metabolic liability and vulnerability compared to other scaffolds, for example as predicted for the lead NSAID analog SSA (please see Supplemental materials).

LogD values computed for compounds in series 1–3 (Tables 1–3) show linear correlation with suppression of Wnt/β-catenin signaling at 10 µM (*r* ^2^ equals 0.74), while at 100 µM drug concentration the analogous correlation is weak (*r* ^2^ equals 0.39). The correlation plot (included in Supplemental materials) suggests that higher logD values are associated with more potent Wnt/β-catenin activities (at 10 µM). Caco-2 cell permeability descriptors computed using Schrödinger software predict that all compounds in this study fall in the range associated with high permeability, except for analogs containing substitution *d* in Fig. 1, which are predicted moderately permeable. StarDrop software tools predict all compounds permeable (human intestinal absorption >30%). While the presented compounds cover a wide range of logD (1.66 to 5.96), nearly all compounds are predicted highly permeable. Further, we found no correlation between the computed logD and cancer cell line data for compounds in series 1–3. For compounds in series 4–5 (Tables 4–5), there was no notable correlation between computed logD values and cancer cell line or Wnt/β-catenin activities.

Thus, computed logD may be correlated with Wnt/β-catenin suppression for analogs in Tables 1–3, however, no analogous correlation was found with cancer cell line activities. Based on in silico predictions cell permeability properties may not be linked to the predicted effect of logD on Wnt/β-catenin activities.

## Conclusions

We synthesized a series of amide libraries from NSAID scaffolds tolfenamic acid, mefenamic acid, flufenamic acid, diclofenac and fenoprofen. These compound sets were evaluated for their anticancer and Wnt/β-catenin signaling activity. Our observations indicated that the benzyl amide derivative of tolfenamic acid **1a** may be a possible candidate for further study relating to the effects of this class on Wnt/β-catenin signaling. It is notable that a number of related analogs show significant inhibition of cell proliferation in the three target cell lines although there appears to be little inhibition of Wnt/β-catenin signaling. As a class, the NSAIDs have been shown to have a variety of cellular activities, and this result is not surprising. We continue to examine the chemical biology of the various NSAID scaffolds with the goal of identifying specific targets that might allow further development of new and improved inhibitors of these targets showing greater potency and selective anticancer activity. A number of analogs from each series are predicted to have acceptable physicochemical properties for consideration in future studies. Among these, analogs **2m**, **5k** and **5o** are highlighted for possible advancement in vivo.

## Electronic supplementary material

Supplementary Information

## Abbreviations

COX: Cyclooxygenase
HLM: Human liver microsome
MW: Molecular weight
NSAIDs: Non-steroidal anti-inflammatory drugs
SSA: Sulindac sulfide amide
