# Supplementary material for: Synthesis and preliminary assessment of the anticancer and Wnt/β-catenin inhibitory activity of small amide libraries of fenamates and profens
Source: Med Chem Res. 2017 Aug 5;26(11):3038–45. doi: 10.1007/s00044-017-2001-z (PMC5656725; doi:10.1007/s00044-017-2001-z)
Supplement: Supplementary file 1 — Supplementary Information [file 44_2017_2001_MOESM1_ESM.docx]

**Supplementary Materials**

**Synthesis and preliminary assessment of the anticancer and Wnt/β-catenin inhibitory activity of small amide libraries of fenamates and profens**

Bini Mathew,^1^ Judith V. Hobrath,^2^ Wenyan Lu,^1^ Yonghe Li,^1^ Robert C. Reynolds^3^

✉ Robert C. Reynolds

[rcr12lkt@uab.edu](mailto:rcr12lkt@uab.edu)

^1^*Drug Discovery Division, Southern Research Institute, 2000 Ninth Avenue South, Birmingham, AL 35205, USA*

*^2^Drug Discovery Unit, College of Life Sciences, University of Dundee, Dundee DD1 5EH, United Kingdom*

*^3^Department of Chemistry and Division of Hematology and Oncology, The University of Alabama at Birmingham, Birmingham, Alabama 35294, USA*

Contents:

I. Metabolic site predictions

II. *In silico* physicochemical property graphs.

III. Experimental details

**I. Metabolic site predictions**

Metabolic sites on selected molecules were predicted using the CYP3A4 metabolism model implemented in StarDrop version 5.5 (from Optibrium Ltd., *www.optibrium.com*). Potential sites of metabolism are labeled on molecule structures and color coded according to the site lability at that position (red: labile, yellow: moderately labile, green: moderately stable, blue: stable). Percentages shown for each site are the predicted percent of products formed due to metabolism occurring at that site. These percent values are given as relative proportions of metabolism at the different sites (and therefore sum up to 100% for each molecule). Thus, percentages between sites may be compared within the same molecule (but not between distinct molecules). Only sites with percentages greater than or equal to 1% are labeled. The metabolic landscape illustrates the lability of each site by vertical bars, height indicating the degree of lability. The composite site lability (CSL) shown is an estimate of the efficiency of metabolism for the entire molecule, calculated from the combined estimated rate of metabolism for all sites of the molecule.

Results of metabolic site predictions are shown for sulindac sulfide amide (SSA), compounds **5o** and **5k**. Computed LogD – MW properties of compounds **5o** and **5k** map to the Golden Triangle region associated with good permeability and metabolic stability properties. Consistently, metabolic site predictions support that compound **5o** contains no high risk labile sites and that it has low CSL value, predicting low efficiency for metabolism. Compound **5k** contains a single labile site at the isopropylamine which may be a target for chemical modifications designed for reducing metabolic liability attributed to this group, while the rest of the scaffold contains only moderately labile and stable sites. Therefore metabolic site computations suggest that compared to SSA for example, both compounds **5o** and **5k** are less vulnerable to metabolic degradation by CYP3A4.

**Metabolic sites and metabolic landscape for sulindac sulfide amide (SSA)**

**Metabolic sites and metabolic landscape for compound 5o.**

**Metabolic sites and metabolic landscape for compound 5k.**

**II. *In silico* physicochemical property graphs.**

Molecular weight versus logD was plotted for all presented compounds, where logD was computed using StarDrop, version 6.2.0 (Optibrium Ltd., *www.optibrium.com*). Compounds that map onto the Golden Triangle area (highlighted in yellow) are shown with labelled data points.

Compounds in series 1 – 3 (listed in Tables 1 – 3) show correlation between their computed logD values and suppression of Wnt/β-catenin signaling activity in HEK293 cells at 10 µM concentration. LogD values correlate with Wnt/β-catenin activities with a correlation coefficient, r of -0.86 and coefficient of determination, r^2^ of 0.74 (20 data points).


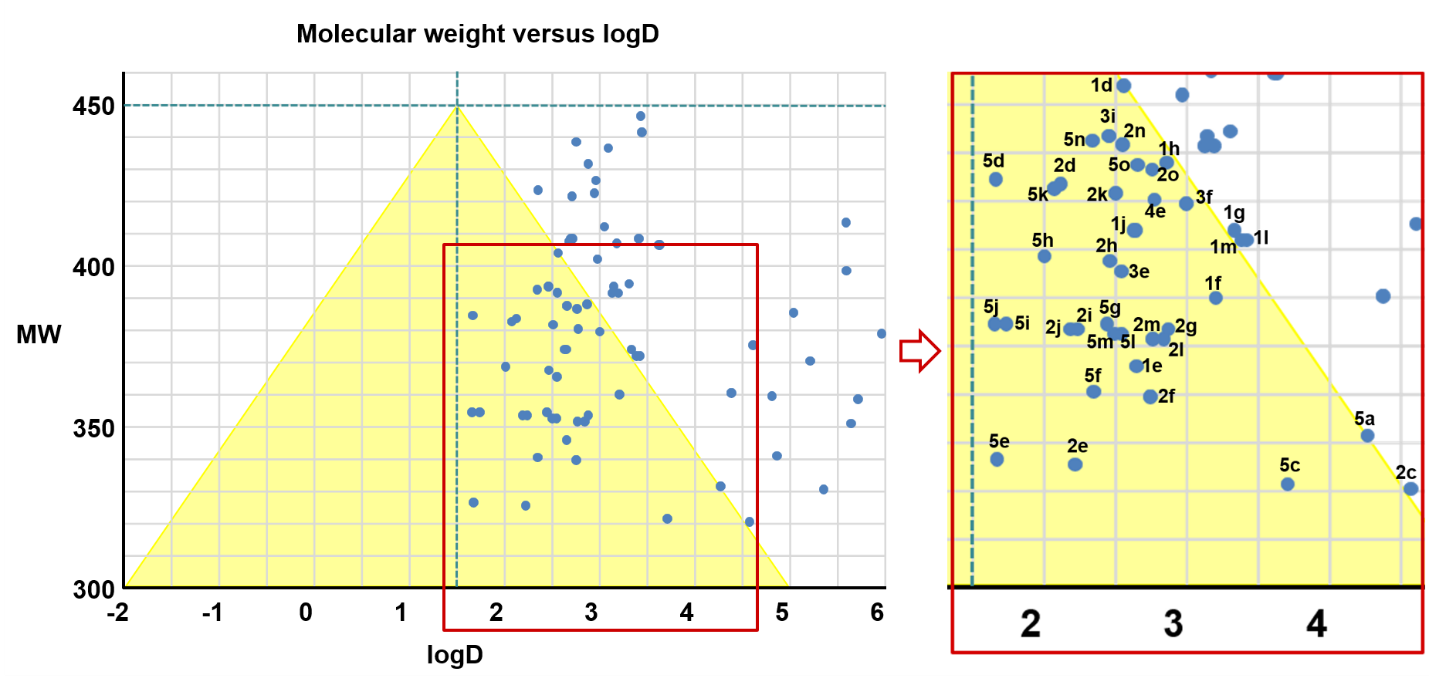


**Molecular weight versus logD for all compounds (Tables 1 – 5).**


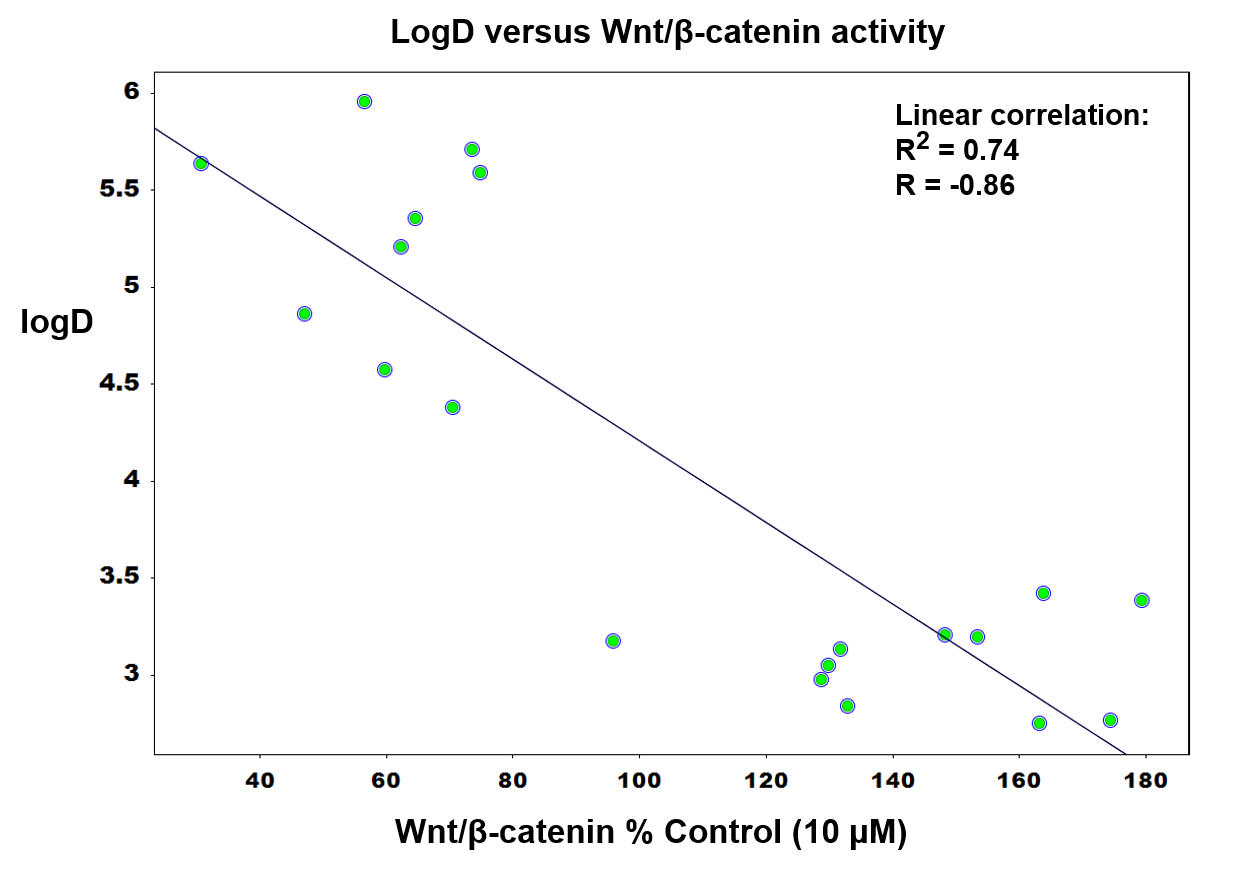


**LogD versus Wnt/β-catenin activity plot for compounds listed in Tables 1 – 3.**

**III. Experimental details**

Anhydrous solvents and reagents from Aldrich were used without further drying. Reactions were monitored by thin-layer chromatography (TLC) on precoated E. Merck silica gel (60F254) plates (0.25 mm) and visualized using UV light (254 nm). Purification of all compounds was carried out by utilizing a Teledyne Isco Combiflash® Rf automated chromatography machine. Melting points, determined with an OptiMelt Automated Melting Point System and, are uncorrected. The exact mass spectral data were obtained with an Agilent LC-MSTOF or with Bruker BIOTOF II by electrospray ionization (ESI). ^1^HNMR spectra were recorded on a Agilent/Varian MR-400 spectrometer operating at 399.930 MHz. Chemical shifts in Me_2_SO-d_6_ are expressed in parts per million downfield from tetramethylsilane (TMS) Chemical shifts (δ) listed for multiplets were measured from the approximate centers, and relative integrals of peak areas agreed with those expected for the assigned structures. Determination of % purity were obtained by HPLC using an Agilent 1100 LC equipped with a diode array UV detector and monitored at multiple wavelengths. ESI-MS spectra were recorded on a BioTof-2 time-of-flight mass spectrometer.

**General method of preparing amides**

(2-(7-Aza-1H-benzotriazole-1-yl)-1,1,3,3-tetramethyluronium hexafluorophosphate), (HATU) (1.2 equivalents) was added to a solution of acid (1 equivalent), the appropriate amine (1.5 equivalents) and DIEA (2 equivalents) in dry acetonitrile (10 mL) at room temperature under argon atmosphere. The reaction mixture was stirred at room temperature for 1-2 h. Solvent was evaporated under reduced pressure and the crude product was purified using a Teledyne Isco Combiflash Rf purification machine to provide the desired amide in excellent yield.

**N-Benzyl-2-((3-chloro-2-methylphenyl)amino)benzamide (1*a*)**

By following general method, the title compound **1*a*** was obtained as a colorless solid in 95% (HPLC purity: 94.2%) yield. mp 115.3-118.1 ^o^C. ^1^H NMR (DMSO, 400 MHz): δ 9.72 (1H, s, NH), 9.16 (1H, t, J = 5.6 Hz, CONH), 7.76 (1H, dd, J = 1.6 Hz, 8.0 Hz, 3’-H), 7.34-7.21 (7H, m, Ph-H, 6-H, 5’-H), 7.17 (1H, t, J = 8.0 Hz, 5-H), 7.13 (1H, dd, J = 1.6 Hz, 8.0 Hz, 4-H), 6.99 (1H, dd, J = 1.2 Hz, 8.8 Hz, 6’-H), 6.84 (1H, td, J = 1.2 Hz, 8.0 Hz, 4’-H), 4.48 (2H, d, J = 6.0 Hz, CH_2_-Ph), 2.24 (3H, s, 2-CH_3_). HRMS calcd for [C_21_H_19_ClN_2_O+H]^+^: 351.12587, Found: 351.12526.

**2-((3-Chloro-2-methylphenyl)amino)-N-(3-phenylpropyl)benzamide (1*b*)**

By following general method, the title compound **1*b*** was obtained as a colorless solid in 94% (HPLC purity: 96.5%) yield. mp 98.7 ^o^C. ^1^H NMR (DMSO, 400 MHz): δ 9.67 (1H, s, NH), 8.60 (1H, t, J = 5.6 Hz, CONH), 7.66 (1H, dd, J = 1.6 Hz, 7.6 Hz, 3’-H), 7.33-7.14 (8H, m, 5-H, 6-H, 5’-H, Ph-H), 7.11 (1H, dd, J = 1.2 Hz, 7.6 Hz, 4-H), 7.00 (1H, dd, J = 0.8 Hz, 8.4 Hz, 6’-H), 6.84 (1H, td, J = 0.8 Hz, 7.6 Hz, 4’-H), 3.27 (2H, q, J = 6.8 Hz, CONHCH_2_), 2.63 (2H, t, J = 7.6 Hz, CH_2_-Ph), 2.25 (3H, s, 2-CH_3_), 1.87-1.79 (2H, m, CH_2_CH_2_-Ph). HRMS calcd for [C_23_H_23_ClN_2_O+H]^+^: 379.15717, Found: 379.15714.

**2-((3-Chloro-2-methylphenyl)amino)-N-(furan-2-ylmethyl)benzamide (1*c*)**

By following general method, the title compound **1*c*** was obtained as a colorless solid in 95% (HPLC purity: 93.2%) yield. mp 92.8-95.0 ^o^C. ^1^H NMR (DMSO, 400 MHz): δ 9.70 (1H, s, NH), 9.08 (1H, t, J = 5.6 Hz, CONH), 7.71 (1H, dd, J = 1.2 Hz, 7.6 Hz, 3’-H), 7.58 (1H, dd, J = 0.8 Hz, 2.0 Hz, 5’’-H), 7.31 (1H, ddd, J = 1.2 Hz, 7.2 Hz, 8.4 Hz, 5’-H), 7.25 (1H, dd, J = 1.2 Hz, 7.6 Hz, 6-H), 7.18 (1H, t, J = 8.0 Hz, 5-H), 7.14 (1H, dd, J = 1.6 Hz, 8.0 Hz, 4-H), 6.98 (1H, dd, J = 0.8 Hz, 8.0 Hz, 6’-H), 6.82 (1H, td, J = 1.2 Hz, 8.4 Hz, 4’-H), 6.39 (1H, dd, J = 2.0 Hz, 3.2 Hz, 4’’-H), 6.29 (1H, dd, J = 0.8 Hz, 3.2 Hz, 3’’-H), 4.46 (2H, d, J = 6.0 Hz, CH_2_-Fu), 2.25 (3H, s, 2-CH_3_). HRMS calcd for [C_19_H_17_ClN_2_O_2_+H]^+^: 341.10513, Found: 341.10539.

**(S)-Ethyl 2-(2-((3-chloro-2-methylphenyl)amino)benzamido)-3-methylbutanoate (1*d*)**

By following general method, the title compound **1*d*** was obtained as a colorless viscous liquid in 94% (HPLC purity: 99.7%) yield. ^1^H NMR (DMSO, 400 MHz): δ 9.24 (1H, s, NH), 8.71 (1H, d, J = 7.2 Hz, CONH), 7.76 (1H, dd, J = 1.6 Hz, 7.6 Hz, 3’-H), 7.34 (1H, ddd, J = 1.6 Hz, 7.6 Hz, 8.8 Hz, 5’-H), 7.22 (1H, dd, J = 1.6 Hz, 7.6 Hz, 6-H), 7.17 (1H, t, J = 8.0 Hz, 5-H), 7.13 (1H, dd, J = 1.6 Hz, 7.6 Hz, 4-H), 6.98 (1H, dd, J = 0.8 Hz, 8.0 Hz, 6’-H), 6.87 (1H, td, J = 1.2 Hz, 8.4 Hz, 4’-H), 4.26 (1H, t, J = 7.6 Hz, CONHCH), 4.18-4.05 (2H, m, COOCH_2_CH_3_), 2.23 (3H, s, 2-CH_3_), 2.21-2.16 (1H, m, CH(CH_3_)_2_), 1.17 (3H, t, J = 7.2 Hz, COOCH_2_CH_3_), 0.98 (3H, d, J = 6.8 Hz, CH(CH_3_)_2_), 0.94 (3H, d, J = 6.8 Hz, CH(CH_3_)_2_). HRMS calcd for [C_21_H_25_ClN_2_O_3_+H]^+^: 389.16265, Found: 389.16256.

**2-((3-Chloro-2-methylphenyl)amino)-N-(3-(dimethylamino)propyl)benzamide (1*e*)**

By following general method, the title compound **1*e*** was obtained as a colorless viscous liquid in 64% (HPLC purity: 100%) yield. ^1^H NMR (DMSO, 400 MHz, 80 ^o^C): δ 9.51 (1H, s, NH), 8.42 (1H, bs, CONH), 7.65 (1H, dd, J = 1.2 Hz, 8.0 Hz, 3’-H), 7.29 (1H, ddd, J = 1.6 Hz, 7.2 Hz, 8.4 Hz, 5’-H), 7.20 (1H, dd, J = 2.0 Hz, 8.0 Hz, 6-H), 7.16 (1H, t, J = 8.0 Hz, 5-H), 7.12 (1H, dd, J = 1.6 Hz, 7.6 Hz, 4-H), 6.93 (1H, dd, J = 1.2 Hz, 8.0 Hz, 6’-H), 6.82 (1H, td, J = 1.2 Hz, 8.0 Hz, 4’-H), 3.33 (2H, q, J = 6.4 Hz, CONHCH_2_), 2.88 (2H, bs, CH_2_N(CH_3_)_2_), 2.60 (6H, s, N(CH_3_)_2_), 2.26 (3H, s, 2-CH_3_), 1.87-1.80 (2H, m, CH_2_CH_2_N(CH_3_)_2_). HRMS calcd for [C_19_H_24_ClN_3_O+H]^+^: 346.16807, Found: 346.16786.

**2-((3-Chloro-2-methylphenyl)amino)-N-(2-(diethylamino)ethyl)benzamide (1*f*)**

By following general method, the title compound **1*f*** was obtained as a colorless viscous liquid in 68% (HPLC purity: 98.9%) yield. ^1^H NMR (DMSO, 400 MHz, 80 ^o^C): δ 9.44 (1H, s, NH), 8.29 (1H, bs, CONH), 7.64 (1H, d, J = 7.2 Hz, 3’-H), 7.29 (1H, t, J = 8.0 Hz, 5’-H), 7.20-7.11 (3H, m, 4-H, 5-H, 6-H), 6.91 (1H, d, J = 8.0 Hz, 6’-H), 6.82 (1H, t, J = 7.6 Hz, 4’-H), 3.43 (2H, q, J = 5.6 Hz, CONHCH_2_), 2.77 (6H, bs, CH_2_N(CH_2_CH_3_)_2_), 2.26 (3H, s, 2-CH_3_), 1.06 (6H, t, J = 6.8 Hz, CH_2_N(CH_2_CH_3_)_2_). HRMS calcd for [C_20_H_26_ClN_3_O+H]^+^: 360.18372, Found: 360.18365.

**2-((3-Chloro-2-methylphenyl)amino)-N-(3-(diethylamino)propyl)benzamide (1*g*)**

By following general method, the title compound **1*g*** was obtained as a colorless solid in 62% (HPLC purity: 100%) yield. mp 125.2 ^o^C. ^1^H NMR (DMSO, 400 MHz, 80 ^o^C): δ 9.47 (1H, s, NH), 8.44 (1H, bt, CONH), 7.65 (1H, dd, J = 1.2 Hz, 8.0 Hz, 3’-H), 7.30 (1H, ddd, J = 1.2 Hz, 7.2 Hz, 8.8 Hz, 5’-H), 7.19 (1H, dd, J = 2.0 Hz, 7.6 Hz, 6-H), 7.16 (1H, t, J = 7.6 Hz, 5-H), 7.12 (1H, dd, J = 2.0 Hz, 7.2 Hz, 4-H), 6.93 (1H, dd, J = 1.2 Hz, 8.0 Hz, 6’-H), 6.83 (1H, td, J = 1.2 Hz, 8.0 Hz, 4’-H), 3.35 (2H, q, J = 6.8 Hz, CONHCH_2_), 3.03 (6H, bs, CH_2_N(CH_2_CH_3_)_2_), 2.26 (3H, s, 2-CH_3_), 1.85-1.81 (2H, m, CH_2_CH_2_N(CH_2_CH_3_)_2_), 1.14 (6H, t, J = 6.8 Hz, CH_2_N(CH_2_CH_3_)_2_). HRMS calcd for [C_21_H_28_ClN_3_O+H]^+^: 374.19937, Found: 374.19839.

**2-((3-Chloro-2-methylphenyl)amino)-N-(2-(diethylamino)ethyl)-N-ethylbenzamide (1*h*)**

By following general method, the title compound **1*h*** was obtained as a colorless viscous liquid in 94% (HPLC purity: 99.1%) yield. ^1^H NMR (DMSO, 400 MHz, 80 ^o^C): δ 7.24 (1H, ddd, J = 1.6 Hz, 7.6 Hz, 8.4 Hz, 5’-H), 7.20 (1H, dd, J = 1.6 Hz, 7.6 Hz, 3’-H), 7.09 (1H, t, J = 8.0 Hz, 5-H), 7.05 (1H, dd, J = 1.6 Hz, 8.0 Hz, 6-H), 6.98 (1H, dd, J = 1.6 Hz, 7.6 Hz, 4-H), 6.95 (1H, s, NH), 6.92 (1H, td, J = 1.2 Hz, 7.6 Hz, 4’-H), 6.86 (1H, d, J = 8.0 Hz, 6’-H), 3.39-3.32 (4H, m, CON(CH_2_CH_3_)CH_2_), 2.45 (2H, t, J = 6.8 Hz, CH_2_N(CH_2_CH_3_)_2_), 2.38 (4H, q, J = 6.8 Hz, CH_2_N(CH_2_CH_3_)_2_), 2.21 (3H, s, 2-CH_3_), 1.03 (3H, t, J = 7.2 Hz, CON(CH_2_CH_3_)CH_2_), 0.84 (6H, t, J = 7.6 Hz, CH_2_N(CH_2_CH_3_)_2_). HRMS calcd for [C_22_H_30_ClN_3_O+H]^+^: 388.21502, Found: 388.21496.

**2-((3-Chloro-2-methylphenyl)amino)-N-(2-(diethylamino)ethyl)-N-methylbenzamide (1*i*)**

By following general method, the title compound **1*i*** was obtained as a colorless viscous liquid in 93% (HPLC purity: 98.8%) yield. ^1^H NMR (DMSO, 400 MHz, 80 ^o^C): δ 7.25-7.05 (5H, m, 5-H, 6-H, 3’-H, 5’-H, NH), 7.01 (1H, d, J = 7.6 Hz, 4-H), 6.89 (1H, td, J = 0.8 Hz, 7.6 Hz, 4’-H), 6.83 (1H, d, J = 8.0 Hz, 6’-H), 3.40 (2H, t, J = 6.8 Hz, CON(CH_3_)CH_2_), 2.93 (3H, s, CON(CH_3_)CH_2_), 2.46 (2H, t, J = 6.4 Hz, CH_2_N(CH_2_CH_3_)_2_), 2.39 (4H, q, J = 6.8 Hz, CH_2_N(CH_2_CH_3_)_2_), 2.21 (3H, s, 2-CH_3_), 0.85 (6H, t, J = 7.2 Hz, CH_2_N(CH_2_CH_3_)_2_). HRMS calcd for [C_21_H_28_ClN_3_O+H]^+^: 374.19937, Found: 374.19964.

**2-((3-Chloro-2-methylphenyl)amino)-N-ethyl-N-(3-(ethylamino)propyl)benzamide (1*j*)**

By following general method, the title compound **1*j*** was obtained as a colorless solid in 68% (HPLC purity: 99.7%) yield. mp 162.2 ^o^C. ^1^H NMR (DMSO, 400 MHz, 80 ^o^C): δ 7.90 (1H, bs, NHCH_2_CH_3_), 7.30-7.222 (2H, m, 3’-H, 5’-H), 7.11-7.04 (2H, m, 5-H, 6-H), 6.98-6.90 (4H, m, 4-H, 4’-H, 6’-H, NH), 3.41 (2H, t, J = 6.8 Hz, CON(CH_2_CH_3_)CH_2_), 3.32 (2H, q, J = 7.2 Hz, CON(CH_2_CH_3_)CH_2_), 2.89-2.80 (4H, m, CH_2_N(CH_2_CH_3_)H), 2.22 (3H, s, 2-CH_3_), 1.85-1.76 (2H, m, CH_2_CH_2_N(CH_2_CH_3_)H), 1.14 (3H, t, J = 7.2 Hz, CON(CH_2_CH_3_)CH_2_), 1.04 (3H, t, J = 7.2 Hz, CH_2_N(CH_2_CH_3_)H). HRMS calcd for [C_21_H_28_ClN_3_O+H]^+^: 374.19937, Found: 374.19934.

**2-((3-Chloro-2-methylphenyl)amino)-N-isopropyl-N-(3-(isopropylamino)propyl)benzamide (1*k*)**

By following general method, the title compound **1*k*** was obtained as a colorless solid in 70% (HPLC purity: 99.6%) yield. mp 177.4 ^o^C. ^1^H NMR (DMSO, 400 MHz, 80 ^o^C): δ 8.00 (1H, bs, NHCH(CH_3_)_2_), 7.28 (1H, t, J = 7.2 Hz, 5’-H), 7.21 (1H, d, J = 7.6 Hz, 3’-H), 7.11-7.04 (2H, m, 5-H, 6-H), 6.98 (1H, t, J = 7.6 Hz, 4’-H), 6.95 (1H, d, J = 8.0 Hz, 4-H), 6.90 (1H, d, J = 8.4 Hz, 6’-H), 6.79 (1H, s, NH), 4.05-3.98 (1H, m, CH_2_N(CH(CH_3_)_2_)H), 3.36 (2H, t, J = 7.2 Hz, CON(CH(CH_3_)_2_)CH_2_), 3.25-3.17 (1H, m, CON(CH(CH_3_)_2_)CH_2_), 2.89 (2H, t, J = 6.8 Hz, CH_2_N(CH(CH_3_)_2_)H), 2.22 (3H, s, 2-CH_3_), 1.90-1.83 (2H, m, CH_2_CH_2_N(CH(CH_3_)_2_)H), 1.20 (6H, d, J = 6.4 Hz, CON(CH(CH_3_)_2_)CH_2_), 1.11 (6H, d, J = 6.8 Hz, CH_2_N(CH(CH_3_)_2_)H). HRMS calcd for [C_23_H_32_ClN_3_O+H]^+^: 402.23067, Found: 402.23095.

**2-((3-Chloro-2-methylphenyl)amino)-N-(2-(piperidin-1-yl)ethyl)benzamide (1*l*)**

By following general method, the title compound **1*l*** was obtained as a colorless viscous liquid in 92% (HPLC purity: 93.5%) yield. ^1^H NMR (DMSO, 400 MHz, 80 ^o^C): δ 9.41 (1H, s, NH), 8.18 (1H, bs, CONH), 7.62 (1H, d, J = 8.0 Hz, 3’-H), 7.27 (1H, t, J = 7.2 Hz, 5’-H), 7.19-7.10 (3H, m, 4-H, 5-H, 6-H), 6.91 (1H, d, J = 8.0 Hz, 6’-H), 6.81 (1H, t, J = 7.2 Hz, 4’-H), 3.38 (2H, q, J = 6.0 Hz, CONHCH_2_), 2.51 (2H, t, J = 6.8 Hz, CONHCH_2_CH_2_), 2.43 (4H, t, J = 5.2 Hz, 2’’-H, 6’’-H), 2.25 (3H, s, 2-CH_3_), 1.51-1.37 (6H, m, 3’’-H, 4’’-H, 5’’-H). HRMS calcd for [C_21_H_26_ClN_3_O+H]^+^: 372.18372, Found: 372.18314.

**(S)-2-((3-Chloro-2-methylphenyl)amino)-N-((1-ethylpyrrolidin-2-yl)methyl)benzamide (1*m*)**

By following general method, the title compound **1*m*** was obtained as a colorless solid in 94% (HPLC purity: 100%) yield. mp 54.7 ^o^C. ^1^H NMR (DMSO, 400 MHz, 80 ^o^C): δ 9.36 (1H, s, NH), 8.46 (1H, bs, CONH), 7.66 (1H, d, J = 8.0 Hz, 3’-H), 7.31 (1H, t, J = 7.6 Hz, 5’-H), 7.19-7.12 (3H, m, 4-H, 5-H, 6-H), 6.91 (1H, d, J = 8.4 Hz, 6’-H), 6.84 (1H, t, J = 8.0 Hz, 4’-H), 3.60-2.80 (7H, m, CONHCH_2_, 1’’-CH_2_CH_3_, 2’’-H, 5’’-H), 2.26 (3H, s, 2-CH_3_), 2.12-1.75 (4H, m, 3’’-H, 4’’-H), 1.19 (3H, bs, 1’’-CH_2_CH_3_). HRMS calcd for [C_21_H_26_ClN_3_O+H]^+^: 372.18372, Found: 372.18333.

**[1,4'-Bipiperidin]-1'-yl(2-((3-chloro-2-methylphenyl)amino)phenyl)methanone (1*n*)**

By following general method, the title compound **1*n*** was obtained as a colorless solid in 91% (HPLC purity: 99.7%) yield. mp 54.3 ^o^C. ^1^H NMR (DMSO, 400 MHz, 80 ^o^C): δ 7.25 (1H, ddd, J = 1.6 Hz, 7.2 Hz, 8.8 Hz, 5’-H), 7.20 (1H, dd, J = 1.6 Hz, 7.6 Hz, 3’-H), 7.12-6.88 (6H, m, 4-H, 5-H, 6-H, 4’-H, 6’-H, NH), 4.02 (2H, bs, 2’’-H_a_, 6’’-H_a_), 2.85 (2H, bs, 2’’-H_b_, 6’’-H_b_), 2.45-2.42 (1H, m, 4’’-H), 2.36 (2H, t, J = 5.6 Hz, 2’’’-H_b_, 6’’’-H_b_), 2.21 (3H, s, 2-CH_3_), 1.68-1.23 (10H, m, 3’’-H, 5’’-H, 3’’’-H, 4’’’-H, 5’’’-H). HRMS calcd for [C_24_H_30_ClN_3_O+H]^+^: 412.21502, Found: 412.21515.

**(2-((3-Chloro-2-methylphenyl)amino)phenyl)(4-(pyridin-4-yl)piperazin-1-yl)methanone (1*o*)**

By following general method, the title compound **1*o*** was obtained as a colorless solid in 94% (HPLC purity: 99.9%) yield. mp 78.1 ^o^C. ^1^H NMR (DMSO, 400 MHz, 80 ^o^C): δ 8.15 (2H, d, J = 6.4 Hz, 2’’’-H, 6’’’-H), 7.28-7.01 (6H, m, 4-H, 5-H, 6-H, 3’-H, 5’-H, NH), 6.91 (1H, t, J = 7.2 Hz, 4’-H), 6.83 (1H, d, J = 8.4 Hz, 6’-H), 6.75 (2H, d, J = 6.4 Hz, 3’’’-H, 5’’’-H), 3.59 (4H, t, J = 4.8 Hz, 2’’-H, 6’’-H), 3.35 (4H, t, J = 5.6 Hz, 3’’-H, 5’’-H), 2.20 (3H, s, 2-CH_3_). HRMS calcd for [C_23_H_23_ClN_4_O+H]^+^: 407.16332, Found: 407.16316.

**N-Benzyl-2-((2,3-dimethylphenyl)amino)benzamide (2*a*)**

By following general method, the title compound **2*a*** was obtained as a colorless solid in 94% (HPLC purity: 96.2%) yield. mp 104.4 ^o^C. ^1^H NMR (DMSO, 400 MHz): δ 9.58 (1H, s, NH), 9.10 (1H, t, J = 6.0 Hz, CONH), 7.72 (1H, dd, J = 1.6 Hz, 8.4 Hz, 3’-H), 7.34-7.22 (6H, m, Ph-H, 5’-H), 7.11 (1H, dd, J = 2.0 Hz, 6.0 Hz, 6-H), 7.06 (1H, t, J = 8.0 Hz, 5-H), 6.93 (1H, dd, J = 2.0 Hz, 6.8 Hz, 4-H), 6.83 (1H, dd, J = 1.2 Hz, 8.4 Hz, 6’-H), 6.74 (1H, td, J = 0.8 Hz, 8.0 Hz, 4’-H), 4.48 (2H, d, J = 5.6 Hz, CH_2_-Ph), 2.26 (3H, s, 2-CH_3_), 2.08 (3H, s, 3-CH_3_). HRMS calcd for [C_22_H_22_N_2_O+H]^+^: 331.18049, Found: 331.18115.

**2-((2,3-Dimethylphenyl)amino)-N-(3-phenylpropyl)benzamide (2*b*)**

By following general method, the title compound **2*b*** was obtained as a colorless solid in 94% (HPLC purity: 97.4%) yield. mp 61.6 ^o^C. ^1^H NMR (DMSO, 400 MHz): δ 9.53 (1H, s, NH), 8.54 (1H, t, J = 5.2 Hz, CONH), 7.63 (1H, dd, J = 1.6 Hz, 8.0 Hz, 3’-H), 7.29-7.03 (8H, m, 5-H, 6-H, 5’-H, Ph-H), 6.91 (1H, dd, J = 2.0 Hz, 6.8 Hz, 4-H), 6.83 (1H, dd, J = 1.2 Hz, 8.4 Hz, 6’-H), 6.73 (1H, td, J = 0.8 Hz, 8.0 Hz, 4’-H), 3.28 (2H, q, J = 7.2 Hz, CONHCH_2_), 2.64 (2H, t, J = 7.6 Hz, CH_2_-Ph), 2.26 (3H, s, 2-CH_3_), 2.09 (3H, s, 3-CH_3_), 1.87-1.80 (2H, m, CH_2_CH_2_-Ph). HRMS calcd for [C_24_H_26_N_2_O+H]^+^: 359.21179, Found: 359.21134.

**2-((2,3-Dimethylphenyl)amino)-N-(furan-2-ylmethyl)benzamide (2*c*)**

By following general method, the title compound **2*c*** was obtained as a colorless solid in 92% (HPLC purity: 100%) yield. mp 107.7-109.4 ^o^C. ^1^H NMR (DMSO, 400 MHz): δ 9.57 (1H, s, NH), 9.01 (1H, t, J = 6.0 Hz, CONH), 7.68 (1H, dd, J = 1.6 Hz, 8.4 Hz, 3’-H), 7.58 (1H, dd, J = 0.8 Hz, 1.6 Hz, 5’’-H), 7.24 (1H, ddd, J = 1.6 Hz, 7.2 Hz, 8.4 Hz, 5’-H), 7.10-7.04 (2H, m, 5-H, 6-H), 6.93 (1H, dd, J = 1.6 Hz, 6.8 Hz, 4-H), 6.81 (1H, dd, J = 0.8 Hz, 8.4 Hz, 6’-H), 6.72 (1H, td, J = 1.2 Hz, 8.0 Hz, 4’-H), 6.40 (1H, dd, J = 1.6 Hz, 3.2 Hz, 4’’-H), 6.29 (1H, dd, J = 0.8 Hz, 3.2 Hz, 3’’-H), 4.46 (2H, d, J = 5.6 Hz, CH_2_-Fu), 2.27 (3H, s, 2-CH_3_), 2.09 (3H, s, 3-CH_3_). HRMS calcd for [C_20_H_20_N_2_O_2_+H]^+^: 321.15975, Found: 321.15931.

**(S)-Ethyl 2-(2-((2,3-dimethylphenyl)amino)benzamido)-3-methylbutanoate (2*d*)**

By following general method, the title compound **2*d*** was obtained as a colorless viscous liquid in 92% (HPLC purity: 100%) yield. ^1^H NMR (DMSO, 400 MHz): δ 9.15 (1H, s, NH), 8.64 (1H, d, J = 7.6 Hz, CONH), 7.73 (1H, dd, J = 1.6 Hz, 8.0 Hz, 3’-H), 7.26 (1H, ddd, J = 1.6 Hz, 7.6 Hz, 8.8 Hz, 5’-H), 7.08-6.91 (3H, m, 4-H, 5-H, 6-H), 6.81 (1H, dd, J = 0.8 Hz, 8.4 Hz, 6’-H), 6.76 (1H, td, J = 1.2 Hz, 8.0 Hz, 4’-H), 4.26 (1H, t, J = 7.6 Hz, CONHCH), 4.19-4.05 (2H, m, COOCH_2_CH_3_), 2.26 (3H, s, 2-CH_3_), 2.24-2.15 (1H, m, CH(CH_3_)_2_), 2.07 (3H, s, 3-CH_3_), 1.18 (3H, t, J = 7.2 Hz, COOCH_2_CH_3_), 0.99 (3H, d, J = 6.8 Hz, CH(CH_3_)_2_), 0.95 (3H, d, J = 6.8 Hz, CH(CH_3_)_2_). HRMS calcd for [C_22_H_28_N_2_O_3_+H]^+^: 369.21727, Found: 369.21779.

**N-(3-(Dimethylamino)propyl)-2-((2,3-dimethylphenyl)amino)benzamide (2*e*)**

By following general method, the title compound **2*e*** was obtained as a colorless viscous liquid in 75% (HPLC purity: 99.0%) yield. ^1^H NMR (DMSO, 400 MHz): δ 9.57 (1H, s, NH), 8.62 (1H, t, J = 5.2 Hz, CONH), 7.64 (1H, dd, J = 1.2 Hz, 8.0 Hz, 3’-H), 7.27-6.91 (4H, m, 4-H, 5-H, 6-H, 5’-H), 6.84 (1H, d, J = 8.4 Hz, 6’-H), 6.74 (1H, t, J = 8.0 Hz, 4’-H), 3.31 (2H, bs, CONHCH_2_), 2.89 (2H, bs, CH_2_N(CH_3_)_2_), 2.61 (6H, s, N(CH_3_)_2_), 2.27 (3H, s, 2-CH_3_), 2.10 (3H, s, 3-CH_3_), 1.86-1.78 (2H, m, CH_2_CH_2_N(CH_3_)_2_). HRMS calcd for [C_20_H_27_N_3_O+H]^+^: 326.22269, Found: 326.22272.

**N-(2-(Diethylamino)ethyl)-2-((2,3-dimethylphenyl)amino)benzamide (2*f*)**

By following general method, the title compound **2*f*** was obtained as a colorless viscous liquid in 47% (HPLC purity: 100%) yield. ^1^H NMR (DMSO, 400 MHz): δ 9.52 (1H, s, NH), 8.55 (1H, bs, CONH), 7.63 (1H, d, J = 7.6 Hz, 3’-H), 7.25 (1H, t, J = 7.2 Hz, 5’-H), 7.09-6.92 (3H, m, 4-H, 5-H, 6-H), 6.82 (1H, d, J = 8.8 Hz, 6’-H), 6.74 (1H, t, J = 7.6 Hz, 4’-H), 3.47 (8H, bs, CONHCH_2_CH_2_N(CH_2_CH_3_)_2_), 2.27 (3H, s, 2-CH_3_), 2.10 (3H, s, 3-CH_3_), 1.09 (6H, bs, CH_2_N(CH_2_CH_3_)_2_). HRMS calcd for [C_21_H_29_N_3_O+H]^+^: 340.23834, Found: 340.23821.

**N-(3-(Diethylamino)propyl)-2-((2,3-dimethylphenyl)amino)benzamide (2*g*)**

By following general method, the title compound **2*g*** was obtained as a colorless solid in 56% (HPLC purity: 100%) yield. mp 131.8 ^o^C. ^1^H NMR (DMSO, 400 MHz): δ 9.53 (1H, s, NH), 8.64 (1H, t, J = 5.6 Hz, CONH), 7.64 (1H, dd, J = 1.6 Hz, 8.0 Hz, 3’-H), 7.26 (1H, ddd, J = 1.6 Hz, 7.6 Hz, 8.4 Hz, 5’-H), 7.07-6.92 (3H, m, 4-H, 5-H, 6-H), 6.84 (1H, dd, J = 1.2 Hz, 8.4 Hz, 6’-H), 6.75 (1H, td, J = 1.2 Hz, 8.0 Hz, 4’-H), 3.35 (2H, q, J = 6.4 Hz, CONHCH_2_), 3.10 (6H, bs, CH_2_N(CH_2_CH_3_)_2_), 2.27 (3H, s, 2-CH_3_), 2.10 (3H, s, 3-CH_3_), 1.92-1.81 (2H, m, CH_2_CH_2_N(CH_2_CH_3_)_2_), 1.16 (6H, t, J = 7.2 Hz, CH_2_N(CH_2_CH_3_)_2_). HRMS calcd for [C_22_H_31_N_3_O+H]^+^: 354.25399, Found: 354.25385.

**N-(2-(Diethylamino)ethyl)-2-((2,3-dimethylphenyl)amino)-N-ethylbenzamide (2*h*)**

By following general method, the title compound **2*h*** was obtained as a colorless viscous liquid in 92% (HPLC purity: 100%) yield. ^1^H NMR (DMSO, 400 MHz, 80 ^o^C): δ 7.17-6.88 (5H, m, 4-H, 5-H, 6-H, 3’-H, 5’-H), 6.79 (1H, td, J = 1.2 Hz, 7.2 Hz, 4’-H), 6.72 (1H, s, NH), 6.68 (1H, d, J = 8.8 Hz, 6’-H), 3.44-3.36 (4H, m, CON(CH_2_CH_3_)CH_2_), 2.51 (2H, t, J = 6.8 Hz, CH_2_N(CH_2_CH_3_)_2_), 2.40 (4H, q, J = 7.2 Hz, CH_2_N(CH_2_CH_3_)_2_), 2.25 (3H, s, 2-CH_3_), 2.06 (3H, s, 3-CH_3_), 1.07 (3H, t, J = 7.2 Hz, CON(CH_2_CH_3_)CH_2_), 0.85 (6H, t, J = 7.2 Hz, CH_2_N(CH_2_CH_3_)_2_). HRMS calcd for [C_23_H_33_N_3_O+H]^+^: 368.26964, Found: 368.27018.

**N-(2-(Diethylamino)ethyl)-2-((2,3-dimethylphenyl)amino)-N-methylbenzamide (2*i*)**

By following general method, the title compound **2*i*** was obtained as a colorless viscous liquid in 92% (HPLC purity: 99.7%) yield. ^1^H NMR (DMSO, 400 MHz, 80 ^o^C): δ 7.17-6.87 (6H, m, 4-H, 5-H, 6-H, 3’-H, 5’-H, NH), 6.77 (1H, td, J = 0.8 Hz, 7.2 Hz, 4’-H), 6.65 (1H, d, J = 8.0 Hz, 6’-H), 3.44 (2H, t, J = 6.8 Hz, CON(CH_3_)CH_2_), 2.96 (3H, s, CON(CH_3_)CH_2_), 2.52 (2H, t, J = 6.4 Hz, CH_2_N(CH_2_CH_3_)_2_), 2.40 (4H, q, J = 7.2 Hz, CH_2_N(CH_2_CH_3_)_2_), 2.26 (3H, s, 2-CH_3_), 2.06 (3H, s, 3-CH_3_), 0.85 (6H, t, J = 7.2 Hz, CH_2_N(CH_2_CH_3_)_2_). HRMS calcd for [C_22_H_31_N_3_O+H]^+^: 354.25399, Found: 354.25394.

**2-((2,3-Dimethylphenyl)amino)-N-ethyl-N-(3-(ethylamino)propyl)benzamide (2*j*)**

By following general method, the title compound **2*j*** was obtained as a colorless viscous liquid in 70% (HPLC purity: 99.4%) yield. ^1^H NMR (DMSO, 400 MHz): δ 8.04 (1H, bs, NHCH_2_CH_3_), 7.24-6.87 (6H, m, 4-H, 5-H, 6-H, 3’-H, 5’-H, NH), 6.85 (1H, t, J = 6.8 Hz, 4’-H), 6.75 (1H, d, J = 8.0 Hz, 6’-H), 3.44 (2H, bs, CON(CH_2_CH_3_)CH_2_), 3.32 (2H, bs, CON(CH_2_CH_3_)CH_2_), 2.85 (4H, bs, CH_2_N(CH_2_CH_3_)H), 2.25 (3H, s, 2-CH_3_), 2.05 (3H, s, 3-CH_3_), 1.82 (2H, bs, CH_2_CH_2_N(CH_2_CH_3_)H), 1.12 (3H, t, J = 7.2 Hz, CON(CH_2_CH_3_)CH_2_), 1.06 (3H, t, J = 7.2 Hz, CH_2_N(CH_2_CH_3_)H). HRMS calcd for [C_22_H_31_N_3_O+H]^+^: 354.25399, Found: 354.25391.

**2-((2,3-Dimethylphenyl)amino)-N-isopropyl-N-(3-(isopropylamino)propyl)benzamide (2*k*)**

By following general method, the title compound **2*k*** was obtained as a colorless solid in 65% (HPLC purity: 98.9%) yield. mp 182.9 ^o^C. ^1^H NMR (DMSO, 400 MHz): δ 8.15 (1H, bs, NHCH_2_CH_3_), 7.23-6.89 (5H, m, 4-H, 5-H, 6-H, 3’-H, 5’-H), 6.86 (1H, td, J = 0.8 Hz, 7.2 Hz, 4’-H), 6.74 (1H, s, NH), 6.70 (1H, d, J = 8.0 Hz, 6’-H), 4.02 (1H, bs, CON(CH(CH_3_)_2_)CH_2_), 3.37 (2H, t, J = 6.4 Hz, CON(CH(CH_3_)_2_)CH_2_), 3.22 (1H, bs, CH_2_N(CH(CH_3_)_2_)H), 2.91 (2H, bs, CH_2_N(CH(CH_3_)_2_)H), 2.25 (3H, s, 2-CH_3_), 2.04 (3H, s, 3-CH_3_), 1.88 (2H, bs, CH_2_CH_2_N(CH(CH_3_)_2_)H), 1.19 (6H, d, J = 6.4 Hz, CON(CH(CH_3_)_2_)CH_2_), 1.13 (6H, d, J = 6.8 Hz, CH_2_N(CH(CH_3_)_2_)H). HRMS calcd for [C_24_H_35_N_3_O+H]^+^: 382.28529, Found: 382.28496.

**2-((2,3-Dimethylphenyl)amino)-N-(2-(piperidin-1-yl)ethyl)benzamide (2*l*)**

By following general method, the title compound **2*l*** was obtained as a colorless viscous liquid in 92% (HPLC purity: 100%) yield. ^1^H NMR (DMSO, 400 MHz): δ 9.47 (1H, s, NH), 8.41 (1H, t, J = 5.6 Hz, CONH), 7.60 (1H, dd, J = 1.6 Hz, 8.0 Hz, 3’-H), 7.23 (1H, ddd, J = 1.6 Hz, 7.2 Hz, 8.8 Hz, 5’-H), 7.08-6.90 (3H, m, 4-H, 5-H, 6-H), 6.81 (1H, dd, J = 0.8 Hz, 8.4 Hz, 6’-H), 6.73 (1H, td, J = 0.8 Hz, 8.0 Hz, 4’-H), 3.36 (2H, q, J = 6.8 Hz, CONHCH_2_), 2.42 (2H, t, J = 6.8 Hz, CONHCH_2_CH_2_), 2.37 (4H, bt, 2’’-H, 6’’-H), 2.26 (3H, s, 2-CH_3_), 2.09 (3H, s, 3-CH_3_), 1.49-1.34 (6H, m, 3’’-H, 4’’-H, 5’’-H). HRMS calcd for [C_22_H_29_N_3_O+H]^+^: 352.23834, Found: 352.23807.

**(S)-2-((2,3-Dimethylphenyl)amino)-N-((1-ethylpyrrolidin-2-yl)methyl)benzamide (2*m*)**

By following general method, the title compound **2*m*** was obtained as a colorless viscous liquid in 94% (HPLC purity: 100%) yield. ^1^H NMR (DMSO, 400 MHz): δ 9.44 (1H, s, NH), 8.42 (1H, t, J = 6.0 Hz, CONH), 7.60 (1H, dd, J = 1.2 Hz, 8.0 Hz, 3’-H), 7.23 (1H, ddd, J = 1.6 Hz, 7.2 Hz, 8.4 Hz, 5’-H), 7.08-6.91 (3H, m, 4-H, 5-H, 6-H), 6.80 (1H, dd, J = 1.2 Hz, 8.4 Hz, 6’-H), 6.73 (1H, td, J = 0.8 Hz, 8.0 Hz, 4’-H), 3.58-2.08 (7H, m, CONHCH_2_, 1’’-CH_2_CH_3_, 2’’-H, 5’’-H), 2.26 (3H, s, 2-CH_3_), 2.09 (3H, s, 3-CH_3_), 1.82-1.57 (4H, m, 3’’-H, 4’’-H), 1.03 (3H, t, J = 7.2 Hz, 1’’-CH_2_CH_3_). HRMS calcd for [C_22_H_29_N_3_O+H]^+^: 352.23834, Found: 352.23883.

**[1,4'-Bipiperidin]-1'-yl(2-((2,3-dimethylphenyl)amino)phenyl)methanone (2*n*)**

By following general method, the title compound **2*n*** was obtained as a colorless viscous liquid in 91% (HPLC purity: 99.5%) yield. ^1^H NMR (DMSO, 400 MHz): δ 7.20-7.15 (2H, m, 3’-H, 5’-H), 7.04-7.00 (2H, m, 5-H, NH), 6.94 (1H, d, J = 7.2 Hz, 6-H), 6.88 (1H, d, J = 7.6 Hz, 4-H), 6.81 (1H, td, J = 0.8 Hz, 7.6 Hz, 4’-H), 6.70 (1H, d, J = 7.6 Hz, 6’-H), 4.60-3.60 (2H, m, 2’’-H_a_, 6’’-H_a_), 2.85 (2H, bs, 2’’-H_b_, 6’’-H_b_), 2.46-2.35 (5H, m, 4’’-H, 2’’’-H, 6’’’-H), 2.25 (3H, s, 2-CH_3_), 2.04 (3H, s, 3-CH_3_), 1.76-1.26 (10H, m, 3’’-H, 5’’-H, 3’’’-H, 4’’’-H, 5’’’-H). HRMS calcd for [C_25_H_33_N_3_O+H]^+^: 392.26964, Found: 392.26961.

**(2-((2,3-Dimethylphenyl)amino)phenyl)(4-(pyridin-4-yl)piperazin-1-yl)methanone (2*o*)**

By following general method, the title compound **2*o*** was obtained as a colorless viscous liquid in 94% (HPLC purity: 99.9%) yield. ^1^H NMR (DMSO, 400 MHz): δ 8.17 (2H, dd, J = 1.6 Hz, 4.8 Hz, 2’’’-H, 6’’’-H), 7.23-6.77 (9H, m, 4-H, 5-H, 6-H, 3’-H, 4’-H, 5’-H, , 3’’’-H, 5’’’-H, NH), 6.63 (1H, d, J = 8.0 Hz, 6’-H), 3.61 (4H, bs, 2’’-H, 6’’-H), 3.39 (4H, bs, 3’’-H, 5’’-H), 2.24 (3H, s, 2-CH_3_), 2.04 (3H, s, 3-CH_3_). HRMS calcd for [C_24_H_26_N_4_O+H]^+^: 387.21794, Found: 387.21798.

**N-Benzyl-2-((3-(trifluoromethyl)phenyl)amino)benzamide (3*a*)**

By following general method, the title compound **3*a*** was obtained as a colorless solid in 94% (HPLC purity: 98.7%) yield. mp 105.1-106.8 ^o^C. ^1^H NMR (DMSO, 400 MHz): δ 9.65 (1H, s, NH), 9.12 (1H, t, J = 5.6 Hz, CONH), 7.73 (1H, dd, J = 1.6 Hz, 8.0 Hz, 3’-H), 7.49-7.20 (11H, m, Ph-H, 2-H, 4-H, 5-H, 6-H, 5’-H, 6’-H), 6.97 (1H, td, J = 1.2 Hz, 8.4 Hz, 4’-H), 4.45 (2H, d, J = 6.4 Hz, CH_2_-Ph). HRMS calcd for [C_21_H_17_F_3_N_2_O+H]^+^: 371.13657, Found: 371.13687.

**N-(3-Phenylpropyl)-2-((3-(trifluoromethyl)phenyl)amino)benzamide (3*b*)**

By following general method, the title compound **3*b*** was obtained as a colorless solid in 93% (HPLC purity: 100%) yield. mp 64.4-65.2 ^o^C. ^1^H NMR (DMSO, 400 MHz): δ 9.56 (1H, s, NH), 8.56 (1H, t, J = 5.6 Hz, CONH), 7.64 (1H, dd, J = 1.6 Hz, 8.0 Hz, 3’-H), 7.48-7.14 (11H, m, Ph-H, 2-H, 4-H, 5-H, 6-H, 5’-H, 6’-H), 6.97 (1H, td, J = 1.2 Hz, 8.0 Hz, 4’-H), 3.24 (2H, q, J = 6.8 Hz, CONHCH_2_), 2.60 (2H, t, J = 7.6 Hz, CH_2_-Ph), 1.83-1.75 (2H, m, CH_2_CH_2_-Ph). HRMS calcd for [C_23_H_21_F_3_N_2_O+H]^+^: 399.16787, Found: 399.16750.

**N-(Furan-2-ylmethyl)-2-((3-(trifluoromethyl)phenyl)amino)benzamide (3*c*)**

By following general method, the title compound **3*c*** was obtained as a colorless solid in 95% (HPLC purity: 98.5%) yield. mp 77.4-78.8 ^o^C. ^1^H NMR (DMSO, 400 MHz): δ 9.64 (1H, s, NH), 9.05 (1H, t, J = 5.6 Hz, CONH), 7.69 (1H, dd, J = 1.2 Hz, 7.6 Hz, 3’-H), 7.56 (1H, dd, J = 0.8 Hz, 2.0 Hz, 5’’-H), 7.50-7.38 (4H, m, 2-H, 5-H, 6-H, 5’-H), 7.34 (1H, dd, J = 1.2 Hz, 8.4 Hz, 6’-H), 7.22 (1H, d, J = 7.2 Hz, 4-H), 6.95 (1H, td, J = 1.2 Hz, 8.0 Hz, 4’-H), 6.38 (1H, dd, J = 2.0 Hz, 3.2 Hz, 4’’-H), 6.27 (1H, d, J = 3.2 Hz, 3’’-H), 4.44 (2H, d, J = 5.2 Hz, CH_2_-Fu). HRMS calcd for [C_19_H_15_F_3_N_2_O_2_+H]^+^: 361.11584, Found: 361.11585.

**(S)-Ethyl 3-methyl-2-(2-((3-(trifluoromethyl)phenyl)amino)benzamido)butanoate (3*d*)**

By following general method, the title compound **3*d*** was obtained as a colorless viscous liquid in 93% (HPLC purity: 99.6%) yield. ^1^H NMR (DMSO, 400 MHz): δ 9.18 (1H, s, NH), 8.72 (1H, d, J = 7.2 Hz, CONH), 7.73 (1H, dd, J = 1.6 Hz, 7.6 Hz, 3’-H), 7.48-7.32 (5H, m, 2-H, 5-H, 6-H, 5’-H, 6’-H), 7.19 (1H, d, J = 7.6 Hz, 4-H), 7.02 (1H, td, J = 1.2 Hz, 8.4 Hz, 4’-H), 4.24 (1H, t, J = 6.8 Hz, CONHCH), 4.16-4.02 (2H, m, COOCH_2_CH_3_), 2.19-2.10 (1H, m, CH(CH_3_)_2_), 1.15 (3H, t, J = 7.2 Hz, COOCH_2_CH_3_), 0.94 (3H, d, J = 7.2 Hz, CH(CH_3_)_2_), 0.90 (3H, d, J = 6.8 Hz, CH(CH_3_)_2_). HRMS calcd for [C_21_H_23_F_3_N_2_O_3_+H]^+^: 409.17335, Found: 409.17415.

**N-(3-(Dimethylamino)propyl)-2-((3-(trifluoromethyl)phenyl)amino)benzamide (3*e*)**

By following general method, the title compound **3*e*** was obtained as a colorless viscous liquid in 78% (HPLC purity: 99.4%) yield. ^1^H NMR (DMSO, 400 MHz, 80 ^o^C): δ 9.45 (1H, s, NH), 8.36 (1H, bs, CONH), 7.64 (1H, dd, J = 1.6 Hz, 8.0 Hz, 3’-H), 7.48-7.28 (5H, m, 2-H, 5-H, 6-H, 5’-H, 6’-H), 7.20 (1H, d, J = 8.0 Hz, 4-H), 6.96 (1H, td, J = 1.2 Hz, 8.0 Hz, 4’-H), 3.28 (2H, q, J = 6.4 Hz, CONHCH_2_), 2.59 (2H, bs, CH_2_N(CH_3_)_2_), 2.38 (6H, s, N(CH_3_)_2_), 1.76-1.69 (2H, m, CH_2_CH_2_N(CH_3_)_2_). HRMS calcd for [C_19_H_22_F_3_N_3_O+H]^+^: 366.17877, Found: 366.17939.

**N-(2-(Diethylamino)ethyl)-2-((3-(trifluoromethyl)phenyl)amino)benzamide (3*f*)**

By following general method, the title compound **3*f*** was obtained as a colorless viscous liquid in 78% (HPLC purity: 99.6%) yield. ^1^H NMR (DMSO, 400 MHz, 80 ^o^C): δ 9.37 (1H, s, NH), 8.27 (1H, bs, CONH), 7.64 (1H, dd, J = 1.6 Hz, 8.0 Hz, 3’-H), 7.48-7.28 (5H, m, 2-H, 5-H, 6-H, 5’-H, 6’-H), 7.19 (1H, d, J = 7.6 Hz, 4-H), 6.97 (1H, td, J = 1.2 Hz, 7.6 Hz, 4’-H), 3.39 (2H, bs, CONHCH_2_), 2.76 (6H, bs, CH_2_N(CH_2_CH_3_)_2_), 1.04 (6H, bs, CH_2_N(CH_2_CH_3_)_2_). HRMS calcd for [C_20_H_24_F_3_N_3_O+H]^+^: 380.19442, Found: 380.19439.

**N-(3-(Diethylamino)propyl)-2-((3-(trifluoromethyl)phenyl)amino)benzamide (3*g*)**

By following general method, the title compound **3*g*** was obtained as a colorless viscous liquid in 78% (HPLC purity: 100%) yield. ^1^H NMR (DMSO, 400 MHz, 80 ^o^C): δ 9.39 (1H, s, NH), 8.41 (1H, bs, CONH), 7.65 (1H, dd, J = 1.6 Hz, 8.0 Hz, 3’-H), 7.48-7.29 (5H, m, 2-H, 5-H, 6-H, 5’-H, 6’-H), 7.20 (1H, d, J = 8.0 Hz, 4-H), 6.98 (1H, td, J = 1.2 Hz, 7.6 Hz, 4’-H), 3.32 (2H, q, J = 6.4 Hz, CONHCH_2_), 3.04 (6H, bs, CH_2_N(CH_2_CH_3_)_2_), 1.89-1.82 (2H, m, CH_2_CH_2_N(CH_2_CH_3_)_2_), 1.15 (6H, t, J = 7.2 Hz, CH_2_N(CH_2_CH_3_)_2_). HRMS calcd for [C_21_H_26_F_3_N_3_O+H]^+^: 394.21007, Found: 394.21019.

**N-(2-(Diethylamino)ethyl)-N-ethyl-2-((3-(trifluoromethyl)phenyl)amino)benzamide (3*h*)**

By following general method, the title compound **3*h*** was obtained as a colorless viscous liquid in 91% (HPLC purity: 98.2%) yield. ^1^H NMR (DMSO, 400 MHz, 80 ^o^C): δ 7.60 (1H, s, NH), 7.37-7.15 (6H, m, 2-H, 5-H, 6-H, 3’-H, 5’-H, 6’-H), 7.09 (1H, td, J = 1.2 Hz, 7.6 Hz, 4’-H), 7.04 (1H, d, J = 8.4 Hz, 4-H), 3.28 (4H, bs, CON(CH_2_CH_3_)CH_2_), 2.40 (2H, t, J = 7.2 Hz, CH_2_N(CH_2_CH_3_)_2_), 2.34 (4H, bs, CH_2_N(CH_2_CH_3_)_2_), 0.97 (3H, t, J = 6.8 Hz, CON(CH_2_CH_3_)CH_2_), 0.83 (6H, t, J = 6.4 Hz, CH_2_N(CH_2_CH_3_)_2_). HRMS calcd for [C_22_H_28_F_3_N_3_O+H]^+^: 408.22572, Found: 408.22581.

**N-(2-(Diethylamino)ethyl)-N-methyl-2-((3-(trifluoromethyl)phenyl)amino)benzamide (3*i*)**

By following general method, the title compound **3*i*** was obtained as a colorless viscous liquid in 92% (HPLC purity: 98.3%) yield. ^1^H NMR (DMSO, 400 MHz, 80 ^o^C): δ 9.45 (1H, s, NH), 7.38-7.05 (8H, m, 2-H, 4-H, 5-H, 6-H, 3’-H, 4’-H, 5’-H, 6’-H), 3.29 (2H, bs, CON(CH_3_)CH_2_), 2.49 (3H, s, CON(CH_3_)CH_2_), 2.42 (2H, t, J = 6.8 Hz, CH_2_N(CH_2_CH_3_)_2_), 2.35 (4H, bs, CH_2_N(CH_2_CH_3_)_2_), 0.83 (6H, t, J = 6.4 Hz, CH_2_N(CH_2_CH_3_)_2_). HRMS calcd for [C_21_H_26_F_3_N_3_O+H]^+^: 394.21007, Found: 394.21027.

**N-Ethyl-N-(3-(ethylamino)propyl)-2-((3-(trifluoromethyl)phenyl)amino)benzamide (3*j*)**

By following general method, the title compound **3*j*** was obtained as a colorless viscous liquid in 70% (HPLC purity: 100%) yield. ^1^H NMR (DMSO, 400 MHz, 80 ^o^C): δ 8.02 (1H, bs, NHCH_2_CH_3_), 7.64 (1H, s, NH), 7.41-7.04 (8H, m, 2-H, 4-H, 5-H, 6-H, 3’-H, 4’-H, 5’-H, 6’-H), 3.37 (2H, bs, CON(CH_2_CH_3_)CH_2_), 3.23 (2H, bs, CON(CH_2_CH_3_)CH_2_), 2.86-2.80 (4H, m, CH_2_N(CH_2_CH_3_)H), 1.83-1.76 (2H, m, CH_2_CH_2_N(CH_3_)H), 1.12 (3H, t, J = 7.6 Hz, CON(CH_2_CH_3_)CH_2_), 0.98 (3H, t, J = 7.2 Hz, CH_2_N(CH_2_CH_3_)H). HRMS calcd for [C_21_H_26_F_3_N_3_O+H]^+^: 394.21007, Found: 394.21116.

**N-Isopropyl-N-(3-(isopropylamino)propyl)-2-((3-(trifluoromethyl)phenyl)amino)benzamide (3*k*)**

By following general method, the title compound **3*k*** was obtained as a colorless solid in 71% (HPLC purity: 100%) yield. mp 105.1 ^o^C. ^1^H NMR (DMSO, 400 MHz, 80 ^o^C): δ 8.00 (1H, bs, NHCH(CH_3_)_2_), 7.58 (1H, s, NH), 7.41-7.12 (7H, m, 2-H, 5-H, 6-H, 3’-H, 4’-H, 5’-H, 6’-H), 7.04 (1H, d, J = 7.2 Hz, 4-H), 3.86 (1H, bs, CH_2_N(CH(CH_3_)_2_)H), 3.30 (2H, t, J = 7.2 Hz, CON(CH(CH_3_)_2_)CH_2_), 3.19-3.13 (1H, m, CON(CH(CH_3_)_2_)CH_2_), 2.84 (2H, bt, CH_2_N(CH(CH_3_)_2_)H), 1.84-1.76 (2H, m, CH_2_CH_2_N(CH(CH_3_)_2_)H), 1.17 (6H, d, J = 6.8 Hz, CON(CH(CH_3_)_2_)CH_2_), 1.03 (6H, d, J = 6.4 Hz, CH_2_N(CH(CH_3_)_2_)H). HRMS calcd for [C_23_H_30_F_3_N_3_O+H]^+^: 422.24137, Found: 422.24149.

**N-(2-(Piperidin-1-yl)ethyl)-2-((3-(trifluoromethyl)phenyl)amino)benzamide (3*l*)**

By following general method, the title compound **3*l*** was obtained as a colorless viscous liquid in 90% (HPLC purity: 99.7%) yield. ^1^H NMR (DMSO, 400 MHz, 80 ^o^C): δ 9.34 (1H, s, NH), 8.16 (1H, bs, CONH), 7.64 (1H, d, J = 7.6 Hz, 3’-H), 7.45 (1H, t, J = 8.4 Hz, 5-H), 7.40-7.27 (4H, m, 2-H, 6-H, 5’-H, 6’-H), 7.18 (1H, d, J = 8.0 Hz, 4-H), 6.97 (1H, t, J = 7.6 Hz, 4’-H), 3.35 (2H, q, J = 6.4 Hz, CONHCH_2_), 3.03 (2H, bs, CONHCH_2_CH_2_), 2.42 (4H, bs, 2’’-H, 6’’-H), 1.50-1.35 (6H, m, 3’’-H, 4’’-H, 5’’-H). HRMS calcd for [C_21_H_24_F_3_N_3_O+H]^+^: 392.19442, Found: 392.19475.

**(S)-N-((1-Ethylpyrrolidin-2-yl)methyl)-2-((3-(trifluoromethyl)phenyl)amino)benzamide (3*m*)**

By following general method, the title compound **3*m*** was obtained as a colorless viscous liquid in 94% (HPLC purity: 100%) yield. ^1^H NMR (DMSO, 400 MHz, 80 ^o^C): δ 9.26 (1H, s, NH), 8.45 (1H, bs, CONH), 7.66 (1H, dd, J = 1.6 Hz, 7.6 Hz, 3’-H), 7.48-7.29 (5H, m, 2-H, 5-H, 6-H, 5’-H, 6’-H), 7.20 (1H, d, J = 7.6 Hz, 4-H), 6.99 (1H, td, J = 1.2 Hz, 8.0 Hz, 4’-H), 3.65-2.80 (7H, m, CONHCH_2_, 1’’-CH_2_CH_3_, 2’’-H, 5’’-H), 2.10-1.65 (4H, m, 3’’-H, 4’’-H), 1.16 (3H, bs, 1’’-CH_2_CH_3_). HRMS calcd for [C_21_H_24_F_3_N_3_O+H]^+^: 392.19442, Found: 392.19478.

**[1,4'-Bipiperidin]-1'-yl(2-((3-(trifluoromethyl)phenyl)amino)phenyl)methanone (3*n*)**

By following general method, the title compound **3*n*** was obtained as a colorless solid in 92% (HPLC purity: 99.1%) yield.

mp 75.1 ^o^C. ^1^H NMR (DMSO, 400 MHz, 80 ^o^C): δ 7.77 (1H, s, NH), 7.39-7.17 (6H, m, 2-H, 5-H, 6-H, 3’-H, 5’-H, 6’-H), 7.10 (1H, td, J = 1.2 Hz, 7.6 Hz, 4’-H), 7.04 (1H, d, J = 7.6 Hz, 4-H), 3.96 (2H, bs, 2’’-H_a_, 6’’-H_a_), 2.65 (2H, bt, 2’’-H_b_, 6’’-H_b_), 2.60-2.30 (5H, bs, 4’’-H, 2’’’-H, 6’’’-H), 1.72-1.15 (10H, m, 3’’-H, 5’’-H, 3’’’-H, 4’’’-H, 5’’’-H). HRMS calcd for [C_24_H_28_F_3_N_3_O+H]^+^: 432.22572, Found: 432.22637.

**(4-(Pyridin-4-yl)piperazin-1-yl)(2-((3-(trifluoromethyl)phenyl)amino)phenyl)methanone (3*o*)**

By following general method, the title compound **3*o*** was obtained as a colorless solid in 92% (HPLC purity: 100%) yield. mp 127.0 ^o^C. ^1^H NMR (DMSO, 400 MHz, 80 ^o^C): δ 8.15 (2H, dd, J = 1.6 Hz, 5.2 Hz, 2’’’-H, 6’’’-H), 7.83 (1H, s, NH), 7.40-7.18 (6H, m, 2-H, 5-H, 6-H, 3’-H, 5’-H, 6’-H), 7.10 (1H, td, J = 0.8 Hz, 7.2 Hz, 4’-H), 7.06 (1H, d, J = 8.0 Hz, 4-H), 6.71 (2H, dd, J = 1.6 Hz, 4.8 Hz, 3’’’-H, 5’’’-H), 3.48 (4H, bs, 2’’-H, 6’’-H), 3.27 (4H, t, J = 5.6 Hz, 3’’-H, 5’’-H). HRMS calcd for [C_23_H_21_F_3_N_4_O+H]^+^: 427.17402, Found: 427.17394.

**N-Benzyl-2-(2-((2,6-dichlorophenyl)amino)phenyl)acetamide (4*a*)**

By following general method, the title compound **4*a*** was obtained as a colorless solid in 93% (HPLC purity: 98.7%) yield. mp 153.7-155.0 ^o^C. ^1^H NMR (DMSO, 400 MHz): δ 8.83 (1H, t, J = 6.0 Hz, CONH), 8.31 (1H, s, NH), 7.51 (2H, d, J = 8.0 Hz, 3-H, 5-H), 7.33-7.20 (6H, m, 3’-H, Ph-H), 7.15 (1H, t, J = 7.6 Hz, 4-H), 7.04 (1H, td, J = 1.6 Hz, 7.6 Hz, 5’-H), 6.85 (1H, td, J = 1.2 Hz, 7.6 Hz, 4’-H), 6.29 (1H, d, J = 7.2 Hz, 6’-H), 4.31 (2H, d, J = 6.0 Hz, CH_2_-Ph), 3.65 (2H, s, 2’-CH_2_). HRMS calcd for [C_21_H_18_Cl_2_N_2_O+H]^+^: 385.08690, Found: 385.08736.

**2-(2-((2,6-Dichlorophenyl)amino)phenyl)-N-(3-phenylpropyl)acetamide (4*b*)**

By following general method, the title compound **4*b*** was obtained as a colorless solid in 93% (HPLC purity: 99.7%) yield. mp 110.0-111.6 ^o^C. ^1^H NMR (DMSO, 400 MHz): δ 8.38 (1H, s, NH), 8.37 (1H, t, J = 5.6 Hz, CONH), 7.50 (2H, d, J = 7.6 Hz, 3-H, 5-H), 7.27-7.13 (7H, m, 3’-H, 4-H, Ph-H), 7.03 (1H, td, J = 0.8 Hz, 7.2 Hz, 5’-H), 6.85 (1H, td, J = 0.8 Hz, 7.6 Hz, 4’-H), 6.28 (1H, d, J = 7.6 Hz, 6’-H), 3.58 (2H, s, 2’-CH_2_), 3.09 (2H, q, J = 7.2 Hz, CONHCH_2_), 2.56 (2H, t, J = 7.6 Hz, CH_2_Ph), 1.75-1.67 (2H, m, CONHCH_2_CH_2_),. HRMS calcd for [C_23_H_22_Cl_2_N_2_O+H]^+^: 413.11820, Found: 413.11891.

**2-(2-((2,6-Dichlorophenyl)amino)phenyl)-N-(furan-2-ylmethyl)acetamide (4*c*)**

By following general method, the title compound **4*c*** was obtained as a colorless solid in 96% (HPLC purity: 96.4%) yield. mp 141.1-143.0 ^o^C. ^1^H NMR (DMSO, 400 MHz): δ 8.80 (1H, t, J = 5.6 Hz, CONH), 8.27 (1H, s, NH), 7.58 (1H, dd, J = 0.8 Hz, 2.0 Hz, 5’’-H), 7.51 (2H, d, J = 7.6 Hz, 3-H, 5-H), 7.19 (1H, dd, J = 1.6 Hz, 8.0 Hz, 3’-H), 7.15 (1H, t, J = 8.0 Hz, 4-H), 7.04 (1H, td, J = 1.6 Hz, 7.6 Hz, 5’-H), 6.84 (1H, td, J = 0.8 Hz, 7.6 Hz, 4’-H), 6.39 (1H, dd, J = 2.0 Hz, 3.6 Hz, 4’’-H), 6.29 (1H, d, J = 7.6 Hz, 6’-H), 6.25 (1H, dd, J = 0.8 Hz, 3.6 Hz, 3’’-H), 4.30 (2H, d, J = 5.2 Hz, CH_2_-Fu), 3.62 (2H, s, 2’-CH_2_). HRMS calcd for [C_19_H_16_Cl_2_N_2_O_2_+H]^+^: 375.06616, Found: 375.06667.

**(S)-Ethyl 2-(2-(2-((2,6-dichlorophenyl)amino)phenyl)acetamido)-3-methylbutanoate (4*d*)**

By following general method, the title compound **4*d*** was obtained as a colorless solid in 95% (HPLC purity: 93.1%) yield. mp 99.0 ^o^C. ^1^H NMR (DMSO, 400 MHz): δ 8.60 (1H, d, J = 8.4 Hz, CONH), 8.05 (1H, s, NH), 7.51 (2H, d, J = 8.0 Hz, 3-H, 5-H), 7.24 (1H, d, J = 7.6 Hz, 3’-H), 7.16 (1H, t, J = 8.0 Hz, 4-H), 7.03 (1H, td, J = 0.8 Hz, 7.6 Hz, 5’-H), 6.84 (1H, t, J = 7.6 Hz, 4’-H), 6.26 (1H, d, J = 8.0 Hz, 6’-H), 4.19 (1H, dd, J = 6.0 Hz, 8.0 Hz, CONHCH), 4.13-4.02 (2H, m, COOCH_2_CH_3_), 3.72 (1H, d, J = 13.2 Hz, 2’-CH_2a_), 3.62 (1H, d, J = 13.6 Hz, 2’-CH_2b_), 2.09-2.01 (1H, m, CH(CH_3_)_2_), 1.15 (3H, t, J = 7.2 Hz, COOCH_2_CH_3_), 0.89 (3H, d, J = 6.8 Hz, CH(CH_3_)_2_), 0.86 (3H, d, J = 6.8 Hz, CH(CH_3_)_2_). HRMS calcd for [C_21_H_24_Cl_2_N_2_O_3_+H]^+^: 423.12367, Found: 423.12387.

**2-(2-((2,6-Dichlorophenyl)amino)phenyl)-N-(3-(dimethylamino)propyl)acetamide (4*e*)**

By following general method, the title compound **4*e*** was obtained as a colorless solid in 94% (HPLC purity: 96.9%) yield. mp 136.7 ^o^C. ^1^H NMR (DMSO, 400 MHz): δ 8.49 (1H, t, J = 6.0 Hz, CONH), 8.30 (1H, s, NH), 7.51 (2H, d, J = 8.0 Hz, 3-H, 5-H), 7.19 (1H, dd, J = 1.6 Hz, 7.6 Hz, 3’-H), 7.16 (1H, t, J = 8.4 Hz, 4-H), 7.05 (1H, td, J = 1.6 Hz, 8.0 Hz, 5’-H), 6.86 (1H, td, J = 1.2 Hz, 7.6 Hz, 4’-H), 6.30 (1H, d, J = 7.2 Hz, 6’-H), 3.58 (2H, s, 2’-CH_2_), 3.14 (2H, q, J = 6.4 Hz, CONHCH_2_), 3.00 (2H, t, J = 8.0 Hz, CONHCH_2_CH_2_CH_2_), 2.72 (6H, s, N(CH_3_)_2_), 1.80-1.73 (2H, m, CON(CH_3_)CH_2_CH_2_). HRMS calcd for [C_19_H_23_Cl_2_N_3_O+H]^+^: 380.12909, Found: 380.12936.

**2-(2-((2,6-Dichlorophenyl)amino)phenyl)-N-(2-(diethylamino)ethyl)acetamide (4*f*)**

By following general method, the title compound **4*f*** was obtained as a colorless solid in 82% (HPLC purity: 98.0%) yield. mp 145.7 ^o^C. ^1^H NMR (DMSO, 400 MHz): δ 8.61 (1H, bs, CONH), 8.12 (1H, s, NH), 7.52 (2H, d, J = 8.0 Hz, 3-H, 5-H), 7.19 (1H, dd, J = 1.6 Hz, 8.0 Hz, 3’-H), 7.17 (1H, t, J = 8.0 Hz, 4-H), 7.05 (1H, td, J = 1.6 Hz, 8.0 Hz, 5’-H), 6.86 (1H, td, J = 0.8 Hz, 7.6 Hz, 4’-H), 6.30 (1H, d, J = 7.6 Hz, 6’-H), 3.61 (2H, s, 2’-CH_2_), 3.43 (2H, bs, CONHCH_2_), 3.14 (6H, bs, CH_2_N(CH_2_CH_3_)_2_), 1.14 (6H, bs, N(CH_2_CH_3_)_2_). HRMS calcd for [C_20_H_25_Cl_2_N_3_O+H]^+^: 394.14474, Found: 394.14586.

**2-(2-((2,6-Dichlorophenyl)amino)phenyl)-N-(3-(diethylamino)propyl)acetamide (4*g*)**

By following general method, the title compound **4*g*** was obtained as a colorless solid in 93% (HPLC purity: 98.2%) yield. mp 56.4 ^o^C. ^1^H NMR (DMSO, 400 MHz): δ 8.51 (1H, t, J = 5.6 Hz, CONH), 8.32 (1H, s, NH), 7.51 (2H, d, J = 8.0 Hz, 3-H, 5-H), 7.20 (1H, dd, J = 1.6 Hz, 7.6 Hz, 3’-H), 7.16 (1H, t, J = 8.4 Hz, 4-H), 7.05 (1H, td, J = 1.6 Hz, 8.0 Hz, 5’-H), 6.86 (1H, td, J = 1.2 Hz, 7.6 Hz, 4’-H), 6.30 (1H, d, J = 7.6 Hz, 6’-H), 3.59 (2H, s, 2’-CH_2_), 3.17 (2H, q, J = 6.4 Hz, CONHCH_2_), 3.10-2.96 (6H, m, CH_2_N(CH_2_CH_3_)_2_), 1.79-1.71 (2H, m, CONHCH_2_CH_2_), 1.12 (6H, t, J = 7.2 Hz, N(CH_2_CH_3_)_2_). HRMS calcd for [C_21_H_27_Cl_2_N_3_O+H]^+^: 408.16039, Found: 408.16080.

**2-(2-((2,6-Dichlorophenyl)amino)phenyl)-N-(2-(diethylamino)ethyl)-N-ethylacetamide (4*h*)**

By following general method, the title compound **4*h*** was obtained as a colorless viscous liquid in 94% (HPLC purity: 97.6%) yield. ^1^H NMR (DMSO, 400 MHz): δ 7.77 (1H, s, NH), 7.52 (2H, d, J = 8.4 Hz, 3-H, 5-H), 7.22 (1H, dd, J = 1.6 Hz, 7.6 Hz, 3’-H), 7.17 (1H, t, J = 8.0 Hz, 4-H), 7.05 (1H, t, J = 8.0 Hz, 5’-H), 6.86 (1H, td, J = 0.4 Hz, 7.2 Hz, 4’-H), 6.28 (1H, d, J = 8.0 Hz, 6’-H), 3.82 (2H, s, 2’-CH_2_), 3.54 (4H, q, J = 7.2 Hz, N(CH_2_CH_3_)_2_), 3.50-3.10 (4H, m, CON(CH_2_CH_3_)CH_2_), 2.54 (2H, bs, CH_2_N(CH_2_CH_3_)_2_), 1.17 (6H, t, J = 7.2 Hz, N(CH_2_CH_3_)_2_), 1.05 (3H, t, J = 7.2 Hz, CON(CH_2_CH_3_)CH_2_). HRMS calcd for [C_22_H_29_Cl_2_N_3_O+H]^+^: 422.17604, Found: 422.17723.

**2-(2-((2,6-Dichlorophenyl)amino)phenyl)-N-(2-(diethylamino)ethyl)-N-methylacetamide (4*i*)**

By following general method, the title compound **4*i*** was obtained as a colorless viscous liquid in 94% (HPLC purity: 97.3%) yield. ^1^H NMR (DMSO, 400 MHz): δ 7.86 (1H, s, NH), 7.51 (2H, d, J = 8.4 Hz, 3-H, 5-H), 7.21 (1H, dd, J = 1.6 Hz, 7.6 Hz, 3’-H), 7.17 (1H, t, J = 8.4 Hz, 4-H), 7.04 (1H, t, J = 7.6 Hz, 5’-H), 6.85 (1H, td, J = 0.8 Hz, 7.6 Hz, 4’-H), 6.29 (1H, d, J = 8.0 Hz, 6’-H), 3.84 (2H, s, 2’-CH_2_), 3.55-3.05 (6H, m, CON(CH_3_)CH_2_, N(CH_2_CH_3_)_2_), 3.16 (3H, s, CON(CH_3_)CH_2_), 2.52 (2H, bs, CH_2_N(CH_2_CH_3_)_2_), 1.04 (6H, bs, N(CH_2_CH_3_)_2_). HRMS calcd for [C_21_H_27_Cl_2_N_3_O+H]^+^: 408.16039, Found: 408.16126.

**2-(2-((2,6-Dichlorophenyl)amino)phenyl)-N-ethyl-N-(3-(ethylamino)propyl)acetamide (4*j*)**

By following general method, the title compound **4*j*** was obtained as a colorless solid in 75% (HPLC purity: 95.3%) yield. mp 131.8 ^o^C. ^1^H NMR (DMSO, 400 MHz): δ 8.16 (1H, bs, NH), 7.86 (1H, s, CH_2_NHCH_2_CH_3_), 7.52 (2H, d, J = 8.0 Hz, 3-H, 5-H), 7.22 (1H, dd, J = 1.6 Hz, 7.2 Hz, 3’-H), 7.17 (1H, t, J = 7.6 Hz, 4-H), 7.05 (1H, td, J = 1.2 Hz, 7.6 Hz, 5’-H), 6.85 (1H, td, J = 1.2 Hz, 7.2 Hz, 4’-H), 6.29 (1H, d, J = 7.6 Hz, 6’-H), 3.82 (2H, s, 2’-CH_2_), 3.51 (2H, q, J = 7.2 Hz, CON(CH_2_CH_3_)CH_2_), 3.36 (2H, t, J = 7.2 Hz, CON(CH_2_CH_3_)CH_2_), 2.98-2.83 (4H, m, CH_2_N(CH_2_CH_3_)H), 1.83-1.76 (2H, m, CH_2_CH_2_N(CH_2_CH_3_)H), 1.18 (3H, t, J = 6.8 Hz, CON(CH_2_CH_3_)CH_2_), 1.12 (3H, t, J = 6.8 Hz, N(CH_2_CH_3_)H). HRMS calcd for [C_21_H_27_Cl_2_N_3_O+H]^+^: 408.16039, Found: 408.16022.

**2-(2-((2,6-Dichlorophenyl)amino)phenyl)-N-isopropyl-N-(3-(isopropylamino)propyl)acetamide (4*k*)**

By following general method, the title compound **4*k*** was obtained as a colorless solid in 75% (HPLC purity: 98.3%) yield. mp 182.8 ^o^C. ^1^H NMR (DMSO, 400 MHz): δ 8.14 (1H, bs, NH), 7.72 (1H, s, CH_2_NHCH(CH_3_)_2_), 7.53 (2H, d, J = 8.0 Hz, 3-H, 5-H), 7.20 (1H, dd, J = 1.2 Hz, 7.6 Hz, 3’-H), 7.18 (1H, t, J = 8.4 Hz, 4-H), 7.05 (1H, td, J = 1.6 Hz, 7.2 Hz, 5’-H), 6.84 (1H, td, J = 1.2 Hz, 7.2 Hz, 4’-H), 6.26 (1H, d, J = 7.2 Hz, 6’-H), 4.40-4.34 (1H, m, CON(CH(CH_3_)_2_)CH_2_), 3.84 (2H, s, 2’-CH_2_), 3.46-3.40 (1H, m, CH_2_N(CH(CH_3_)_2_)H), 3.24 (2H, t, J = 7.6 Hz, CON(CH(CH_3_)_2_)CH_2_), 2.89 (2H, t, J = 7.2 Hz, CH_2_N(CH_2_CH_3_)H), 1.84-1.76 (2H, m, CH_2_CH_2_N(CH(CH_3_)_2_)H), 1.17 (6H, d, J = 6.8 Hz, CON(CH(CH_3_)_2_)CH_2_), 1.13 (6H, d, J = 6.4 Hz, N(CH(CH_3_)_2_)H). HRMS calcd for [C_23_H_31_Cl_2_N_3_O+H]^+^: 436.19169, Found: 436.19087.

**2-(2-((2,6-Dichlorophenyl)amino)phenyl)-N-(2-(piperidin-1-yl)ethyl)acetamide (4*l*)**

By following general method, the title compound **4*l*** was obtained as a colorless solid in 96% (HPLC purity: 100%) yield. mp 160.4 ^o^C. ^1^H NMR (DMSO, 400 MHz): δ 8.47 (1H, bs, CONH), 8.21 (1H, s, NH), 7.51 (2H, d, J = 8.4 Hz, 3-H, 5-H), 7.19 (1H, dd, J = 1.2 Hz, 7.6 Hz, 3’-H), 7.16 (1H, t, J = 8.4 Hz, 4-H), 7.04 (1H, td, J = 1.6 Hz, 7.6 Hz, 5’-H), 6.85 (1H, td, J = 1.2 Hz, 7.2 Hz, 4’-H), 6.29 (1H, d, J = 8.0 Hz, 6’-H), 3.60 (2H, s, 2’-CH_2_), 3.20-2.50 (8H, m, CONHCH_2_CH_2_, 2’’-H, 6’’-H), 1.59 (4H, bs, 3’’-H, 5’’-H), 1.43 (2H, bs, 4’’-H). HRMS calcd for [C_21_H_25_Cl_2_N_3_O+H]^+^: 406.14474, Found: 406.14559.

**(S)-2-(2-((2,6-Dichlorophenyl)amino)phenyl)-N-((1-ethylpyrrolidin-2-yl)methyl)acetamide (4*m*)**

By following general method, the title compound **4*m*** was obtained as a colorless viscous liquid in 94% (HPLC purity: 99.8%) yield. ^1^H NMR (DMSO, 400 MHz): δ 8.19 (1H, s, NH), 7.51 (2H, d, J = 8.4 Hz, 3-H, 5-H), 7.20 (1H, dd, J = 1.6 Hz, 7.6 Hz, 3’-H), 7.16 (1H, t, J = 8.4 Hz, 4-H), 7.04 (1H, td, J = 1.2 Hz, 8.0 Hz, 5’-H), 6.85 (1H, t, J = 7.2 Hz, 4’-H), 6.29 (1H, d, J = 8.0 Hz, 6’-H), 3.62 (2H, s, 2’-CH_2_), 3.58-2.80 (7H, m, CONHCH_2_, 1’’-CH_2_CH_3_, 2’’-H, 5’’-H), 2.15-1.48 (4H, m, 3’’-H, 4’’-H), 1.10 (3H, bs, 1’’-CH_2_CH_3_). HRMS calcd for [C_21_H_25_Cl_2_N_3_O+H]^+^: 406.14474, Found: 406.14513.

**1-([1,4'-Bipiperidin]-1'-yl)-2-(2-((2,6-dichlorophenyl)amino)phenyl)ethanone (4*n*)**

By following general method, the title compound **4*n*** was obtained as a colorless solid in 92% (HPLC purity: 98.9%) yield.

mp 134.2 ^o^C. ^1^H NMR (DMSO, 400 MHz): δ 7.82 (1H, s, NH), 7.52 (2H, d, J = 8.0 Hz, 3-H, 5-H), 7.20 (1H, dd, J = 1.2 Hz, 7.6 Hz, 3’-H), 7.17 (1H, t, J = 7.6 Hz, 4-H), 7.03 (1H, td, J = 1.6 Hz, 8.0 Hz, 5’-H), 6.84 (1H, td, J = 1.2 Hz, 7.2 Hz, 4’-H), 6.26 (1H, d, J = 7.2 Hz, 6’-H), 4.44-4.41 (1H, m, 2’’-H_a_), 4.22-4.16 (1H, m, 6’’-H_a_), 3.83 (1H, d, J = 14.4 Hz, 2’-CH_2a_), 3.77 (1H, d, J = 14.8 Hz, 2’-CH_2b_), 3.10-3.05 (1H, m, 2’’-H_b_), 2.67-2.32 (6H, m, 6’’-H_b_, 4’’-H, 2’’’-H, 6’’’-H), 1.76-1.15 (10H, m, 3’’-H, 5’’-H, 3’’’-H, 4’’’-H, 5’’’-H). HRMS calcd for [C_24_H_29_Cl_2_N_3_O+H]^+^: 446.17604, Found: 446.17661.

**2-(2-((2,6-Dichlorophenyl)amino)phenyl)-1-(4-(pyridin-4-yl)piperazin-1-yl)ethanone (4*o*)**

By following general method, the title compound **4*o*** was obtained as a colorless solid in 92% (HPLC purity: 99.1%) yield.

mp 214.4 ^o^C. ^1^H NMR (DMSO, 400 MHz): δ 8.17 (2H, dd, J = 1.6 Hz, 4.8 Hz, 2’’’-H, 6’’’-H), 7.75 (1H, s, NH), 7.52 (2H, d, J = 8.4 Hz, 3-H, 5-H), 7.23 (1H, dd, J = 1.6 Hz, 7.6 Hz, 3’-H), 7.17 (1H, t, J = 7.6 Hz, 4-H), 7.04 (1H, td, J = 1.2 Hz, 7.6 Hz, 5’-H), 6.85 (1H, td, J = 1.2 Hz, 7.2 Hz, 4’-H), 6.82 (2H, dd, J = 1.6 Hz, 4.8 Hz, 3’’’-H, 5’’’-H), 6.27 (1H, d, J = 7.6 Hz, 6’-H), 3.87 (2H, s, 2’-CH_2_), 3.80 (2H, t, J = 5.2 Hz, 2’’-H), 3.65 (2H, t, J = 5.2 Hz, 6’’-H), 3.38-3.33 (4H, m, 3’’-H, 5’’-H). HRMS calcd for [C_23_H_22_Cl_2_N_4_O+H]^+^: 441.12434, Found: 441.12361.

**N-Benzyl-2-(3-phenoxyphenyl)propanamide (5*a*)**

By following general method, the title compound **5*a*** was obtained as a colorless viscous liquid in 89% (HPLC purity: 90.2%) yield. ^1^H NMR (DMSO, 400 MHz): δ 8.48 (1H, t, J = 6.0 Hz, NH), 7.39-6.84 (14H, m, Ar-H, Ph-H), 4.29-4.18 (2H, m, CH_2_-Ph), 3.66 (1H, q, J = 7.2 Hz, 1’-CH(CH_3_)CO), 1.33 (3H, d, J = 6.8 Hz, 1’-CH(CH_3_)CO). HRMS calcd for [C_22_H_21_NO_2_+H]^+^: 332.16451, Found: 332.16490.

**2-(3-Phenoxyphenyl)-N-(3-phenylpropyl)propanamide (5*b*)**

By following general method, the title compound **5*b*** was obtained as a colorless solid in 95% (HPLC purity: 98.3%) yield. mp 61.3-62.9 ^o^C. ^1^H NMR (DMSO, 400 MHz): δ 7.97 (1H, t, J = 5.6 Hz, NH), 7.36-6.82 (14H, m, Ar-H, Ph-H), 3.58 (1H, q, J = 7.2 Hz, 1’-CH(CH_3_)CO), 3.09-2.94 (2H, m, CONHCH_2_), 2.47 (2H, t, J = 8.0 Hz, CH_2_Ph), 1.66-1.58 (2H, m, CONHCH_2_CH_2_), 1.29 (3H, d, J = 7.2 Hz, 1’-CH(CH_3_)CO). HRMS calcd for [C_24_H_25_NO_2_+H]^+^: 360.19581, Found: 360.19570.

**N-(Furan-2-ylmethyl)-2-(3-phenoxyphenyl)propanamide (5*c*)**

By following general method, the title compound **5*c*** was obtained as a colorless viscous liquid in 92% (HPLC purity: 95.4%) yield. ^1^H NMR (DMSO, 400 MHz): δ 8.42 (1H, t, J = 6.0 Hz, NH), 7.53 (1H, dd, J = 0.8 Hz, 2.0 Hz, 5’’-H), 7.40-6.82 (9H, m, Ar-H, Ph-H), 6.35 (1H, dd, J = 2.0 Hz, 3.2 Hz, 4’’-H), 6.09 (1H, dd, J = 0.8 Hz, 3.2 Hz, 3’’-H), 4.22 (2H, d, J = 5.6 Hz, CH_2_-Fu), 3.63 (1H, q, J = 7.2 Hz, 1’-CH(CH_3_)CO), 1.31 (3H, d, J = 7.2 Hz, 1’-CH(CH_3_)CO). HRMS calcd for [C_20_H_19_NO_3_+H]^+^: 322.14377, Found: 322.14389.

**(2S)-Ethyl 3-methyl-2-(2-(3-phenoxyphenyl)propanamido)butanoate (5*d*)**

By following general method, the title compound **5*d*** was obtained as a colorless viscous liquid in 95% (HPLC purity: 95.6%) yield. ^1^H NMR (DMSO, 400 MHz): δ 8.23 (1H, d, J = 8.0 Hz, NH), 7.40-6.82 (9H, m, Ar-H, Ph-H), 4.15-3.96 (3H, m, COOCH_2_CH_3_, CONHCH), 3.81 (1H, q, J = 7.2 Hz, 1’-CH(CH_3_)CO), 2.07-1.92 (1H, m, CH(CH_3_)_2_), 1.30 (3H, d, J = 7.2 Hz, 1’-CH(CH_3_)CO), 1.18 (3H, t, J = 7.2 Hz, COOCH_2_CH_3_), 0.88 (3H, d, J = 6.8 Hz, CH(CH_3_)_2_), 0.74 (3H, d, J = 6.8 Hz, CH(CH_3_)_2_). HRMS calcd for [C_22_H_27_NO_4_+H]^+^: 370.20128, Found: 370.20114.

**N-(3-(Dimethylamino)propyl)-2-(3-phenoxyphenyl)propanamide (5*e*)**

By following general method, the title compound **5*e*** was obtained as a colorless solid in 91% (HPLC purity: 100%) yield. mp 129.3 ^o^C. ^1^H NMR (DMSO, 400 MHz): δ 8.10 (1H, t, J = 6.0 Hz, CONH), 7.41-6.83 (9H, m, Ar-H, Ph-H), 3.57 (1H, q, J = 6.8 Hz, 1’-CH(CH_3_)CO), 3.05 (2H, q, J = 6.8 Hz, CONHCH_2_), 2.89 (2H, t, J = 8.0 Hz, CONHCH_2_CH_2_CH_2_), 2.69 (6H, s, N(CH_3_)_2_), 1.73-1.65 (2H, m, CONHCH_2_CH_2_), 1.31 (3H, d, J = 7.2 Hz, 1’-CH(CH_3_)CO). HRMS calcd for [C_20_H_26_N_2_O_2_+H]^+^: 327.20670, Found: 327.20764.

**N-(2-(Diethylamino)ethyl)-2-(3-phenoxyphenyl)propanamide (5*f*)**

By following general method, the title compound **5*f*** was obtained as a colorless viscous liquid in 93% (HPLC purity: 98.1%) yield. ^1^H NMR (DMSO, 400 MHz): δ 8.13 (1H, bs, NH), 7.42-6.83 (9H, m, Ar-H, Ph-H), 3.58 (1H, q, J = 7.2 Hz, 1’-CH(CH_3_)CO), 3.42-2.78 (8H, m, CONHCH_2_CH_2_N(CH_2_CH_3_)_2_), 1.31 (3H, d, J = 7.2 Hz, 1’-CH(CH_3_)CO), 1.06 (6H, bs, N(CH_2_CH_3_)_2_). HRMS calcd for [C_21_H_28_N_2_O_2_+H]^+^: 341.22235, Found: 341.22213.

**N-(3-(Diethylamino)propyl)-2-(3-phenoxyphenyl)propanamide (5*g*)**

By following general method, the title compound **5*g*** was obtained as a colorless viscous liquid in 93% (HPLC purity: 98.5%) yield. ^1^H NMR (DMSO, 400 MHz): δ 8.11 (1H, t, J = 6.0 Hz, NH), 7.42-6.82 (9H, m, Ar-H, Ph-H), 3.57 (1H, q, J = 6.8 Hz, 1’-CH(CH_3_)CO), 3.15-2.87 (8H, m, CONHCH_2_CH_2_CH_2_N(CH_2_CH_3_)_2_), 1.74-1.64 (2H, m, CONHCH_2_CH_2_), 1.31 (3H, d, J = 7.2 Hz, 1’-CH(CH_3_)CO), 1.10 (6H, t, J = 7.6 Hz, N(CH_2_CH_3_)_2_). HRMS calcd for [C_22_H_30_N_2_O_2_+H]^+^: 355.23800, Found: 355.23782.

**N-(2-(Diethylamino)ethyl)-N-ethyl-2-(3-phenoxyphenyl)propanamide (5*h*)**

By following general method, the title compound **5*h*** was obtained as a colorless viscous liquid in 94% (HPLC purity: 97.3%) yield. ^1^H NMR (DMSO, 400 MHz): δ 7.41-6.85 (9H, m, Ar-H, Ph-H), 4.05 (1H, q, J = 6.8 Hz, 1’-CH(CH_3_)CO), 3.50-2.54 (10H, m, CON(CH_2_CH_3_)CH_2_CH_2_N(CH_2_CH_3_)_2_), 1.27 (3H, d, J = 6.4 Hz, 1’-CH(CH_3_)CO), 1.08 (6H, bs, N(CH_2_CH_3_)_2_), 0.91 (3H, t, J = 7.2 Hz, CON(CH_2_CH_3_)CH_2_). HRMS calcd for [C_23_H_32_N_2_O_2_+H]^+^: 369.25365, Found: 369.25385.

**N-(2-(Diethylamino)ethyl)-N-methyl-2-(3-phenoxyphenyl)propanamide (5*i*)**

By following general method, the title compound **5*i*** was obtained as a colorless viscous liquid in 95% (HPLC purity: 98.0%) yield. ^1^H NMR (DMSO, 400 MHz): δ 7.41-6.84 (9H, m, Ar-H, Ph-H), 4.07 (1H, q, J = 7.2 Hz, 1’-CH(CH_3_)CO), 3.50-2.98 (6H, m, CON(CH_3_)CH_2_, N(CH_2_CH_3_)_2_), 2.91 (3H, s, CON(CH_3_)CH_2_), 2.50-2.32 (2H, m, CH_2_N(CH_2_CH_3_)_2_), 1.26 (3H, d, J = 6.8 Hz, 1’-CH(CH_3_)CO), 1.08 (3H, bs, N(CH_2_CH_3_)_2_), 0.89 (3H, bs, N(CH_2_CH_3_)_2_). HRMS calcd for [C_22_H_30_N_2_O_2_+H]^+^: 355.23800, Found: 355.23825.

**N-Ethyl-N-(3-(ethylamino)propyl)-2-(3-phenoxyphenyl)propanamide (5*j*)**

By following general method, the title compound **5*j*** was obtained as a colorless viscous liquid in 75% (HPLC purity: 97.7%) yield. ^1^H NMR (DMSO, 400 MHz): δ 8.13 (1H, bs, NH), 7.42-6.84 (9H, m, Ar-H, Ph-H), 4.07 (1H, q, J = 6.8 Hz, 1’-CH(CH_3_)CO), 3.45-3.03 (4H, m, CON(CH_2_CH_3_)CH_2_CH_2_CH_2_), 2.92-2.70 (4H, m, CON(CH_2_CH_3_)CH_2_, N(CH_2_CH_3_)H), 1.79-1.63 (2H, m, CON(CH_2_CH_3_)CH_2_CH_2_), 1.29 (3H, d, J = 6.4 Hz, 1’-CH(CH_3_)CO), 1.13 (3H, t, J = 7.2 Hz, N(CH_2_CH_3_)H), 0.94 (3H, t, J = 7.2 Hz, CON(CH_2_CH_3_)CH_2_). HRMS calcd for [C_22_H_30_N_2_O_2_+H]^+^: 355.23800, Found: 355.23825.

**N-Isopropyl-N-(3-(isopropylamino)propyl)-2-(3-phenoxyphenyl)propanamide (5*k*)**

By following general method, the title compound **5*k*** was obtained as a colorless viscous liquid in 77% (HPLC purity: 98.0%) yield. ^1^H NMR (DMSO, 400 MHz): δ 8.12 (1H, bs, NH), 7.41-6.86 (9H, m, Ar-H, Ph-H), 4.24-4.14 (1H, m, CON(CH(CH_3_)_2_)CH_2_), 4.15 (1H, q, J = 7.2 Hz, 1’-CH(CH_3_)CO), 3.27-3.12 (3H, m, CH_2_N(CH(CH_3_)_2_)H), 2.83 (2H, t, J = 7.6 Hz, CON(CH(CH_3_)_2_)CH_2_), 1.78-1.69 (2H, m, CON(CH(CH_3_)_2_)CH_2_CH_2_), 1.28 (3H, d, J = 6.8 Hz, 1’-CH(CH_3_)CO), 1.19 (6H, d, J = 6.4 Hz, N(CH(CH_3_)_2_)H), 1.13 (3H, d, J = 6.8 Hz, CON(CH(CH_3_)_2_)CH_2_), 0.70 (3H, d, J = 6.4 Hz, CON(CH(CH_3_)_2_)CH_2_). HRMS calcd for [C_24_H_34_N_2_O_2_+H]^+^: 383.26930, Found: 383.26942.

**2-(3-Phenoxyphenyl)-N-(2-(piperidin-1-yl)ethyl)propanamide (5*l*)**

By following general method, the title compound **5*l*** was obtained as a colorless viscous liquid in 94% (HPLC purity: 98.2%) yield. ^1^H NMR (DMSO, 400 MHz): δ 7.80 (1H, t, J = 5.2 Hz, NH), 7.41-6.81 (9H, m, Ar-H, Ph-H), 3.58 (1H, q, J = 7.2 Hz, 1’-CH(CH_3_)CO), 3.17-3.04 (2H, m, CONHCH_2_), 2.29-2.21 (6H, m, CONHCH_2_CH_2_, 2’’-H, 6’’-H), 1.45-1.31 (6H, m, 3’’-H, 4’’-H, 5’’-H), 1.28 (3H, d, J = 6.8 Hz, 1’-CH(CH_3_)CO). HRMS calcd for [C_22_H_28_N_2_O_2_+H]^+^: 353.22235, Found: 353.22225.

**N-(((S)-1-Ethylpyrrolidin-2-yl)methyl)-2-(3-phenoxyphenyl)propanamide (5*m*)**

By following general method, the title compound **5*m*** was obtained as a colorless viscous liquid in 92% (HPLC purity: 98.2%) yield. ^1^H NMR (DMSO, 400 MHz): δ 8.31 (1H, bs, CONH), 7.41-6.84 (9H, m, Ar-H, Ph-H), 3.62 (1H, q, J = 7.2 Hz, 1’-CH(CH_3_)CO), 3.58-2.80 (7H, m, CONHCH_2_, 1’’-CH_2_CH_3_, 2’’-H, 5’’-H), 2.10-1.44 (4H, m, 3’’-H, 4’’-H), 1.31 (3H, d, J = 6.8 Hz, 1’-CH(CH_3_)CO), 1.08 (3H, bs, 1’’-CH_2_CH_3_). HRMS calcd for [C_22_H_28_N_2_O_2_+H]^+^: 353.22235, Found: 353.22303.

**1-([1,4'-Bipiperidin]-1'-yl)-2-(3-phenoxyphenyl)propan-1-one (5*n*)**

By following general method, the title compound **5*n*** was obtained as a colorless viscous liquid in 57% (HPLC purity: 97.5%) yield. ^1^H NMR (DMSO, 400 MHz): δ 7.40-6.82 (9H, m, Ar-H, Ph-H), 4.46-4.34 (1H, m, 2’’-H_a_), 4.10 (1H, q, J = 7.6 Hz, 1’-CH(CH_3_)CO), 3.96-3.86 (1H, m, 6’’-H_a_), 2.94-2.85 (1H, m, 2’’-H_b_), 2.46-2.20 (6H, m, 6’’-H_b_, 4’’-H, 2’’’-H, 6’’’-H), 1.68-0.95 (10H, m, 3’’-H, 5’’-H, 3’’’-H, 4’’’-H, 5’’’-H), 1.24 (3H, d, J = 6.4 Hz, 1’-CH(CH_3_)CO). HRMS calcd for [C_25_H_32_N_2_O_2_+H]^+^: 393.25365, Found: 393.25378.

**2-(3-Phenoxyphenyl)-1-(4-(pyridin-4-yl)piperazin-1-yl)propan-1-one (5*o*)**

By following general method, the title compound **5*o*** was obtained as a colorless viscous liquid in 93% (HPLC purity: 99.5%) yield. ^1^H NMR (DMSO, 400 MHz): δ 8.16 (2H, dd, J = 1.6 Hz, 5.2 Hz, 2’’’-H, 6’’’-H), 7.36-6.85 (9H, m, Ar-H, Ph-H), 6.76 (2H, dd, J = 1.6 Hz, 4.8 Hz, 3’’’-H, 5’’’-H), 4.18 (1H, q, J = 6.8 Hz, 1’-CH(CH_3_)CO), 3.70-2.72 (8H, m, 2’’-H, 3’’-H, 5’’-H, 6’’-H), 1.28 (3H, d, J = 6.8 Hz, 1’-CH(CH_3_)CO). HRMS calcd for [C_24_H_25_N_3_O_2_+H]^+^: 388.20195, Found: 388.20226.
